# Simulations of Structure and Morphology in Photoreactive Polymer Blends under Multibeam Irradiation

**DOI:** 10.1021/acs.jpcc.1c09993

**Published:** 2022-04-06

**Authors:** Nannan Ding, Ian D. Hosein

**Affiliations:** Department of Biomedical and Chemical Engineering, Syracuse University, Syracuse, New York 13244, United States

## Abstract

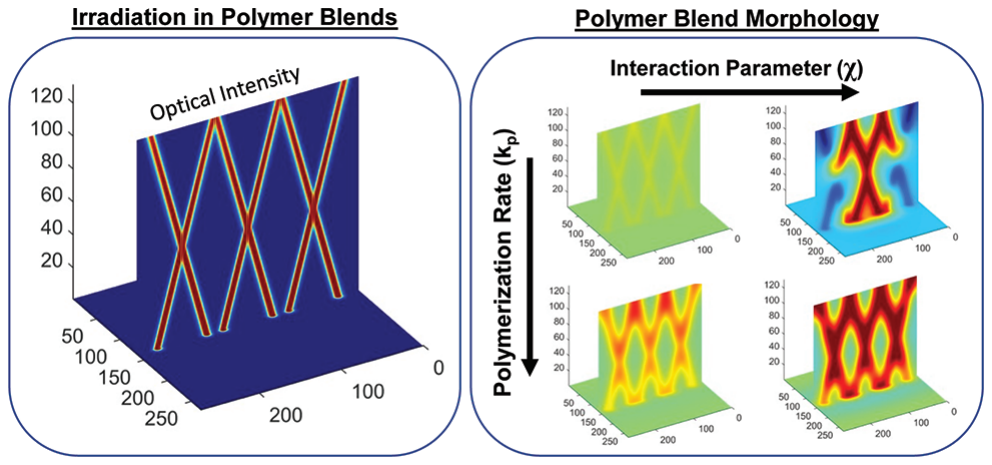

We present a theoretical study of the organization of photoreactive polymer blends under irradiation by multiple arrays of intersecting optical beams. In a simulated medium possessing an integrated intensity-dependent refractive index, optical beams undergo self-focusing and reduced divergence. A corresponding intensity-dependent increase in molecular weight induces polymer blend instability and consequent phase separation, whereby the medium can evolve into an intersecting waveguide lattice structure, comprising high refractive index cylindrical cores and a surrounding low refractive index medium (cladding). We conduct simulations for two propagation angles and a range of thermodynamic, kinetic, and polymer blend parameters to establish correlations to structure and morphology. We show that spatially correlated structures, namely, those that have a similar intersecting three-dimensional (3D) pattern as the arrays of intersecting optical beams, are achieved via a balance between the competitive processes of photopolymerization rate and phase separation dynamics. A greater intersection angle of the optical beams leads to higher correlations between structures and the optical beam pattern and a wider parameter space that achieves correlated structures. This work demonstrates the potential to employ complex propagating light patterns to create 3D organized structures in multicomponent photoreactive soft systems.

## Introduction

Directing the organization of polymer blends into new structures and morphologies is an important approach toward the creation of soft materials with functional properties.^1^ Specifically, producing polymer materials from reactive blends enables the concurrent assembly of complex structures over the course of polymerization. The reaction-induced increase in molecular weight can render the blend thermodynamically immiscible, thereby driving polymerization-induced phase separation (PIPS).^2−7^ This leads to interesting morphologies including droplet phases, cocontinuous phases, and even quasi-ordered systems, yet generally over the macroscale they are randomly organized.^8^ Driving the polymerization reaction via light irradiation (i.e., photopolymerization) with either UV or visible light has also proven quite successful as a means for controlling the polymerization reaction and thereby the structure evolution.^9^ Additionally, the use of three-dimensional (3D) constructive optical fields (i.e., holographic polymerization) is a further advance to pattern phase-separating systems into extremely well-ordered morphologies.^10^

Most recently, photoreactive polymer blends have been organized using single arrays of optical beams transmitted through photoreactive monomer blend media that are sensitized to undergo photoinitiation at the optical beam’s particular wavelength.^1,11^ The optical beams are observed to undergo a self-focusing nonlinearity, associated with a photochemically induced increase in refractive index, which counters the optical beam’s natural divergence.^12−17^ The dynamic balance of self-focusing and divergence leads to self-trapped beams, which propagate divergence-free, or beams with reduced divergence transmitted through the polymer blend medium. As a result, the polymer blend morphology evolves via spatially controlled phase separation uniformly along its depth, with one polymer component in the regions of the optical beams (i.e., regions of irradiation) and the other in the nonirradiated regions surrounding it, effectively organizing the blend into the same pattern as the ensemble of transmitted optical beams.^1,11,15^ The structure and morphology of the blend can then be controlled via light intensity,^15^ optical beam size and interspacing in an array,^18^ formulation composition and component molecular weight,^19−21^ and component volume fractions.^19^

To advance the complexity of the organized structures, there is interest in examining structure formation in polymer blend media irradiated with multiple, nonparallel arrays of optical beams. Several studies have employed the propagation of single or multiple (intersecting and interleaving) nonlinear optical beams in single-component photopolymerizable media that, in turn, inscribe polymer structure with two-dimensional (2D) and 3D symmetries and yield interesting material functionalities.^18,22−27^ Other experimental works employed light-induced self-writing (LISW) and light-induced fiber/rod growth to fabricate 2D and 3D structures.^12,28−32^ The deployment of multiple arrays of optical beams in polymer blends is thus intriguing for the organization of multicomponent media into more complex structures.

Toward this end, theoretical insight into the formation of morphology and understanding of the determinative factors entailed in forming polymer blend structure would be extremely informative for the pursuit of synthesis studies, especially as the parameter space for blends has additional degrees of freedom (e.g., volume fraction, polymer miscibility, molecular weight, refractive index difference, etc.). We have previously established a multiphysics simulation framework for photoreactive polymer blends that combines the multiple phenomena entailed in the self-trapping/self-focusing of an optical beam, photopolymerization kinetics, and phase separation in the proximity of the optical beam, whereby polymer blend morphology could be theoretically predicted and mapped over a range of polymer blends, growth kinetics, and thermodynamic parameters.^33^ In this study, we employ this multiphysics simulation framework to now theoretically investigate the evolution of structure and morphology in photoreactive polymer blends under irradiation with arrays of intersecting beams. The study herein leverages our established multiphysics framework to now particularly examine the implication of the construction of complex morphologies and structures using multiple, intersecting optical beams and the dependencies of the blend and processing parameters enabling the optical beams to pattern binary phase morphology into a 3D structure. The advancements in this study entail the examination of the formation of a more complex structure (rather than examining local phase separation along a single optical beam), assessment of the dependence of spatially varying optical intensities in 3D owing to the intersection of the beams, and a close elucidation of the dependence of the blend and processing parameters particularly on the formation of correlated structures (relative of the optical beams), rather than only the physical mechanisms of phase separation examined previously. The simulated optical beams undergo self-focusing in the photochemically induced nonlinearity and inscribe their intersecting structure in the medium, which, in turn, undergoes polymerization-induced phase separation. As a result, a lattice structure can form, which consists of the intersecting arrays of cylindrical cores comprising one polymer component, surrounded by a common medium comprising the second polymer component. By tuning both the reaction rate and the thermodynamic miscibility of the blend, we find that a balance between reaction kinetics and blend stability enables the binary phase morphology to evolve into the same pattern as the arrays of optical beams and that final morphology depends on the orientation of the optical beams. We describe and quantitatively correlate the interplay of polymerization rate, interaction parameter, molecular weight, component volume fraction, and beam orientation to the final structure. Waveguide arrays can form from a range of polymer blends (in terms of their miscibility), indicating the capability to control morphology and structure through polymerization rate, blend composition, and beam orientation. New insights provided in this study include (1) concurrent modulation of multiple intersecting beams, (2) assessment of their modulation both via photopolymerization-induced nonlinearity and intersection, (3) the effect of optical beam propagation angle on morphology, and (4) quantitative analysis of the spatial correlation between polymer blend morphology/structure and the optical beams, both in final morphologies and during their evolution. These insights can inform on the experimental processing of photoreactive polymer blends into such 3D intersecting waveguide structures with well-defined morphologies.

## Methods

### Theoretical Framework

Full details of the multiphysics simulation framework can be found in our previous study.^33^ Brief descriptions of the different phenomena are described herein.

#### Propagation of Arrays of Optical Beams

Propagation of microscale cylindrically shaped, nonpolarized optical beams (Gaussian profile) of diameter *d* through the medium was computed via the beam propagation method (BPM),^34−36^ as implemented in the RSoft Environment and BPM implementation (Synopsys, BeamProp). Light simulated herein represents propagation of a coherent source (i.e., a laser beam) at a wavelength of 633 nm. The refractive index, *n*, of the medium was calculated according to the weighted mixture rule^37^
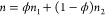
1where *n*_1_ and *n*_2_ are the refractive indices of polymer 1 and polymer 2, respectively, and ϕ (also referred to herein as φ) is defined as the volume fraction for polymer 1. We selected refractive index values to emulate those for trimethylolpropane triacrylate (TMPTA, *n*_1_ = 1.474) and a dimethylsiloxane oligomer (*n*_2_ = 1.446) employed in our previous work.^16,38^ The maximal refractive index difference for TMPTA is ∼Δ*n* = 0.007, which is typical for acrylate systems, and 1 order of magnitude less and more than this (i.e., 0.07 and 0.0007) were explored herein to examine structure dependency on Δ*n*. The high index difference of 0.07, while greater than that found in common polymers, was explored more so to examine the dependence of Δ*n* on morphology from a theoretical standpoint.

#### Photopolymerization Kinetics

The high refractive index polymer (component 1) was reactive, while the low index polymer (component 2) was nonreactive. The degree of polymerization of polymer 1, *X_n_*, is expressed as a function of the light intensity (*I*) and polymerization rate constant (*k*_p_) according to a rate model suitable for mono- and polyfunctional monomers^39−41^
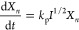
2Absorbance losses and photoinitiation efficiency were excluded from consideration, owing to the small cell depth employed herein, whereby absorbance would not have a significant impact on morphology. Likewise, photoinitiation efficiency is easily offset by intensity to achieve similar dosages. The refractive index (*n*) related to the change in the degree of polymerization was calculated by
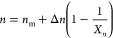
3where *n*_m_ is the refractive index of the monomer, and Δ*n* is the refractive index at saturation (i.e., fully polymerized). Equations 1–3 establish the intensity-dependent increase in the medium refractive index, whose change depends on the time-integrated total irradiation intensity (i.e., dosage).^12,42^ These equations extend the model proposed previously for intensity-dependent refractive index increases^43^ by now incorporating the polymerization kinetic model associated with the increase in refractive index.

#### Phase Separation

To simulate the diffusion of the polymer components, we employed the Cahn–Hilliard equation for a two-component medium^44^
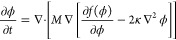
4where *M* is the mobility, κ is the gradient energy coefficient, and *f*(φ) is the Flory–Huggins (FH) free-energy equation for a binary polymer blend^44^
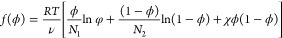
5where *k*_B_ is the Boltzmann constant, *T* is the temperature in Kelvin (298 K), ν is the volume of a lattice size, *N*_1_ (*N*_1_ = *N*(*t*) = *X_n_*, increases over time) and *N*_2_ (fixed) are the degrees of polymerization for polymer 1 and 2, respectively, and χ is the interaction parameter. We employed a polymerization-dependent mobility to simulate reduced diffusion dynamics with an increased degree of polymerization in the system^44^
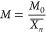
6where  is the average molecular weight of polymer 1, and *M*_0_ is the mobility of the monomer estimated as^45^
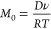
7where *D* is the diffusivity estimated as 5 × 10^–13^ m^2^/s for the polymers,^46−50^ and ν is selected as the molar volume of water, 1.8 × 10^–5^ m^–3^/mol. The gradient energy coefficient was estimated using the random phase approximation^51^
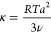
8where *a* is the monomer size, estimated to be 0.54 nm, based on the radius of gyration (*R*_g_) for TMPTA.^52^ We assume in this model that the molecular weight dependence of mobility is such that large polymer chains are essentially immobile, and hence the molecular weight distribution does not significantly change from the diffusion of such molecules. Likewise, large molecular weights of polymers and their low mobility would also inhibit an influx of the monomer of polymer 1 into, as well as expulsion of polymer 2 out of, these regions. Hence, it is also a reasonable assumption that the mobility of the entire system depends primarily on the mobility of the growing polymer 1, which can be represented by a molecular weight-dependent mobility, *M*_0_, for the entire system. These assumptions certainly hold true for cross-linking systems, such as TMPTA, based on our previous experimental studies, which show arrested phase separation with very high conversion in the irradiated regions.^15,18^ Finally, the Cahn–Hilliard equation using Neumann boundary conditions was solved numerically through discretization in both space and time and through the use of spectral methods.^53,54^

### Simulation Box

Calculations were carried out in a 3D volume (*x*, *y*, *z*) spatially discretized into 270 × 270 × 275 or 270 × 270 × 140 points for optical beams propagating at ±15 and ±30°, respectively. The simulation cell length (z direction) was selected to observe a single incidence of intersection among the optical beams and their propagation thereafter. The cell lengths were selected to end just at the second instance of beam intersections. Optical path lengths of the ±15 and ±30° optical beams were 161.6 and 284.7 μm, respectively, along their slanted directions. The Rayleigh length (*z*_r_), at which the beam width increases by √2, for the simulated 8 μm beams at a wavelength of 633 nm is ∼317 μm.

The spacing between points in all dimensions was normalized to represent a 1 μm unit distance to simulate the physical scenario of a microscale optical beam and morphology evolution at the microscale. Propagation of the optical beams was set at angles relative to the *z* axis. Herein, all 3D distributions for both polymer concentration and light intensity are visually displayed as *yz*-plane slices that cut through the center of the simulation cell. We use transparent boundary conditions in the simulations, allowing light to leave the simulation cell at its end (*z*) as well as the sides (*x* and *y*).

### Simulation Design and Procedure

We selected the beam propagation angles of ±15 and ±30° as they (1) are practical angles for which individual optical beams may propagate in a medium without interaction and (2) simulate waveguide formation at angles that can be experimentally produced, as in previous works.^23,55,56^ Under the ideal case of pure and fully cured cylindrical waveguides of polymer 1 phase (*n*_1_ = 1.474 + 0.007) and a pure polymer 2 surroundings (*n*_2_ = 1.446), the collection ranges for waveguides oriented at 15 and 30° would be (10.3, 35.1) and (33.5, 65.1). Hence, combinations of two optical beams, i.e., ±15 and ±30°, would not result in transmitted light interacting or transferring from waveguide to the other that intersects with it. Especially during the waveguide evolution, where light propagation is nonlinear (i.e., optical soliton propagation) with lower refractive index differences than the theoretical maximum, they are expected to pass through one another without interaction. Beam propagation at angles below ∼±8° would begin to interact, as the lower boundary for their acceptance range is common at ∼0°. Angles that are too large (e.g., 45°), which are theoretically feasible to simulate, are impractical from a synthesis standpoint because the maximum angle of the waveguide is limited by Snell’s law. For example, at a maximum incident angle of 90° incoming irradiation (impractical but to demonstrate the point) for an incident beam on a photocurable resin of TMPTA, the waveguide angle would be ∼42°. Incident angles of ∼23 and ∼48° could produce waveguides of 15 and 30°. Hence, these selected angles are well spaced from one another as well as from the practical boundaries from the perspectives of interactions and practical synthesis.

The simulation begins with a medium of a uniformly mixed blend of a reactive monomer (polymer 1) and an inert linear polymer chain (polymer 2). Two arrays of optical beams are launched symmetrically at angles of ±θ, either 15 or 30° in this study. The arrays consisted of cylindrical beams of 8 μm diameter spaced 80 μm apart at the entrance face of the cell (*z* = 0). Each array consisted of three beams, hence a total of six optical beams in each simulation.

Simulation of optical beam propagation and the evolution of polymerization and phase separation was achieved through a temporal loop, for which each iteration sequentially calculates the effect of the following phenomena: (1) propagating two arrays of optical beams through the mixture (BPM method); (2) calculating the intensity- and position-dependent increases in the polymerization degree of polymer 1; (3) calculating the FH free energy of mixing; (4) time-stepping the diffusion of the polymers; and (5) calculating the updated 3D refractive index profile. This updated refractive index profile is used as input for the beginning of the next loop. The time step for each loop was considered as constituting a 100 ms duration (i.e., irradiation by the optical beams), which is the approximate time for a polymerization event in photoinitiated media.^12^ The time step for diffusion was 10 ms, resulting in 10 time steps per iteration (i.e., 10 ms × 10 = 100 ms). The blends are thermodynamically stable at the onset of the simulation, as shown elsewhere.^15,33^

### Parameter Variation

We explored different angles of propagation, polymerization rates via the rate constant (*k*_p_), miscibility via the interaction parameter (χ), different polymerization degrees for polymer 2 (*N*_2_), relative polymer volume fractions, as well as refractive index difference (Δ*n*). All simulations were run for a total simulated duration of 10 s, which was more than sufficient for the phase morphology to evolve to its fullest extent. We used normalized intensities of 2 and 4 for the propagation of 30 and 15° arrays, the latter for which is higher owing to the need for greater power to elicit sufficient polymerization and phase separation over its greater depth (i.e., along the *z* dimension). Doubling the intensity is rationalized owing to the ∼2× greater cell length and corresponding greater divergence of the beam to decouple depth dependence and to enable meaningful comparisons between the two propagation angles. Note that because polymerization rate depends on *I*^0.5^, the polymerization kinetics are not significantly different, and thus the morphologies can be compared. Table 1 summarizes the parameters explored in this study. Based on experiments on curing kinetics with lasers,^57^ the dimensionless values of *k*_p_ used herein would correspond to realistic steady-state polymerization rate constants of 0.1–10 mol·L^–1^·s^–1^, and the intensity *I* would correspond to realistic values of ∼0.8 and 1.6 W/cm^2^ for propagating beams of 30 and 15°, respectively.

**Table 1:** Parameters Explored in This Simulation Study

parameter	values
refractive index difference, Δ*n*	0.0007, 0.007, 0.07
refractive index of polymer 1, *n*_1_	1.474
refractive index of polymer 1, *n*_2_	1.446
volume fraction of polymer 1, φ_1_	0.25, 0.50, 0.75
interaction parameter, χ	0.5, 0.75. 1. 1.25, 1.5
molecular weight of polymer 2, *N*_2_	50, 5000
polymerization rate constant, *k*_p_	0.1, 1, 10, 100
angle of beam propagation, θ_wg_	±15 or ±30 (symmetric)
beam intensity, *I*	2, 4 (nominal)
beam diameter, *d*	8 μm
beam spacing, *S*	80 μm
beam wavelength, λ	0.633 μm
total simulation time	10 s

### Quantitative Spatial Correlations between Optical Beam Pattern and Blend Morphology

To quantify the spatial correlation between the optical beam patterns and the final structures, we calculated the correlation coefficient (*r*) value between the 3D distributions of polymer 1 (*P*_1_) and the optical intensity (*I*)
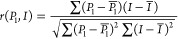
9

## Results and Discussion

In this study, we examined two arrays of optical beams propagated through the medium at two different angles and over a range of polymer blend parameters, polymerization rates, and thermodynamic miscibility. Examining structure and morphology formation in polymer blends irradiated with two arrays constitutes a simple system to reveal informative principles on the correlations between the synthetic, kinetic, and thermodynamic parameters and polymer structures. We are also particularly interested in the morphologies formed using two arrays at non-normal (i.e., slanted) orientations relative to the surface normal (i.e., *z* axis in our simulation box) for their potential application in wide-angle light-collecting structures associated with rotational shifts in the acceptance cones of the cylindrical waveguides, enabling greater collection of light at greater incident angles, as demonstrated in single-component media.^23,32,55,56,58,59^ In particular, the use of polymer blends would enable the core-cladding refractive index difference to be greater than achievable in single-component media, thereby widening the acceptance range (i.e., numerical aperture).

### Optical Beam Propagation, Self-Focusing, and Spatial Profiles during Photopolymerization

**Figure 1:**
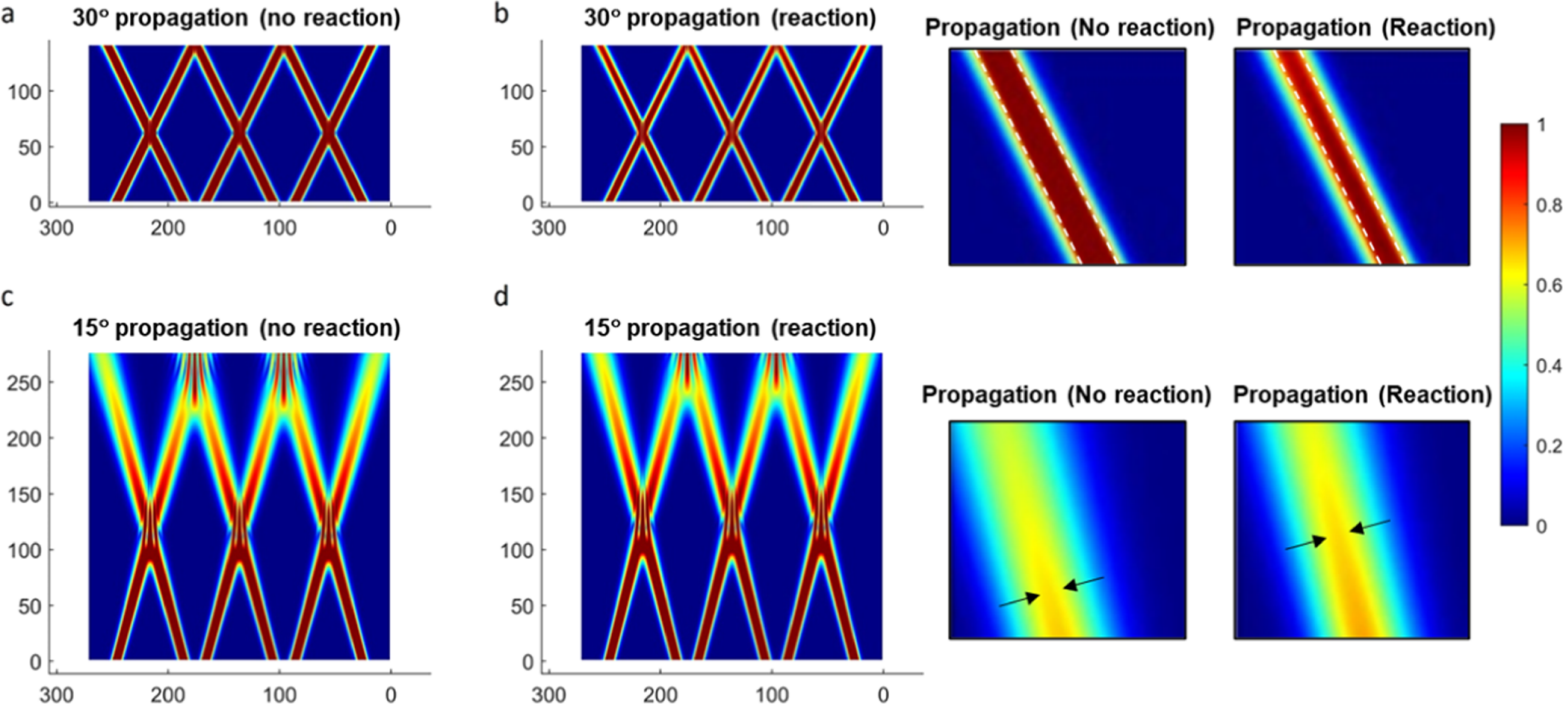
Spatial intensity profiles of the initial *t* = 0 s (a, c) and (b, d) final (*t* = 10 s) propagation profiles of two arrays of optical beams (three beams per array) oriented at (a) ±30° and (b) ±15°. Parameters: φ = 0.5, χ = 1.5, *k*_p_ = 100, Δ*n* = 0.007, *N*_2_ = 50. The insets show higher-magnification images of the initial and final optical beam profiles at the end of the simulation cell. White dashed lines and black arrows serve as guides to observe the modulation and reduction in divergence of the intensity profile associated with the self-focusing component of nonlinear optical propagation.

Figure 1 shows slices of the 3D spatial profiles of the free space propagation of the optical beams prior to and after any induction of the photochemical reaction. The orientation angles of the optical beams were all along a common *yz*-plane and hence coplanar in this sense. All beams naturally diverge as they propagate over space. However, upon the subsequent increase in the refractive index through intensity-dependent polymerization in the regions of irradiation, the beams are observed to undergo different degrees of reduced divergence, owing to the presence of the self-focusing nonlinearity. The ±30° propagating beams become slightly narrower in their intensity distribution, and the ±15° propagating beams show an increase in intensity at their centers. The ±30° propagating beams are simulated for ∼1/2 of the length as the ±15° beams and hence would not show as much divergence. The 8 μm beams propagating at ±30° are approximately the same width at their exit point of the cell, whereas their propagation at ±15° results in ∼11.5 μm beam widths. Hence, the path lengths of the optical beams are not long enough to suffer any significant divergence, but some degree of modulation is observed. It should be noted that the spatial intensities shown in Figure 1 comprise optical beams that experience the simulated optical nonlinearity associated with photopolymerization-induced changes in refractive index. While modulations to their profile are mild, the irradiation with propagating optical beams is distinguished from the fixed optical patterns that are produced by such methods as holography, especially as beams herein can also be incoherent in experimental realizations, which is not possible for fixed coherent holographic fields. Concisely, the 3D optical patterns shown in Figure 1 are different from those established through holography, the latter of which would experimentally entail using several coherent light sources to establish constructive interference. Nevertheless, the spatial intensity profiles in Figure 1 confirm that the optical beam intensities and their profiles can be well preserved over the course of the simulation, so they may drive polymerization and phase separation. The intersecting optical pattern could be produced using two optical sources (which can also be incoherent) and a common photomask.^23^ Experimentally, incoherent sources may also be used to generate these optical beam patterns in contrast to holography.

### Temporal Evolution of Concentration, Molecular Weight, Beam Intensity, and Refractive Index

**Figure 2:**
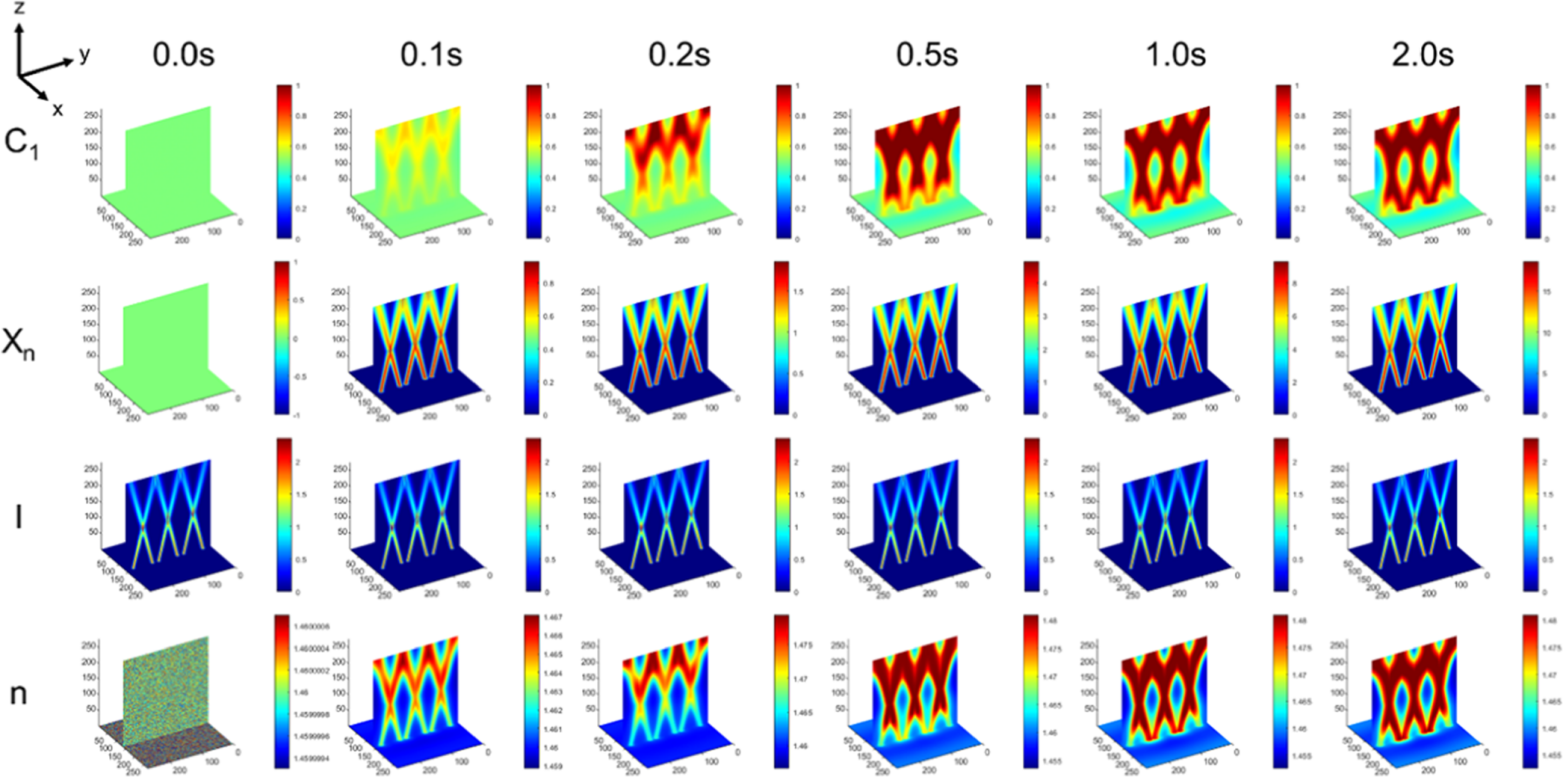
Time evolution in a blend that leads to an intersecting structure, as revealed by concentration (*C*_1_), molecular weight (*X_n_*), beam intensity (*I*), and refractive index (*n*) in the first 2 s of simulation. Parameters: φ = 0.5, χ = 1, *k*_p_ = 10, *N*_2_ = 50, θ = ±15°. System selected for its visually apparent spatial correlation between polymer morphology (*C*_1_) and the optical profile (*I*).

**Figure 3:**
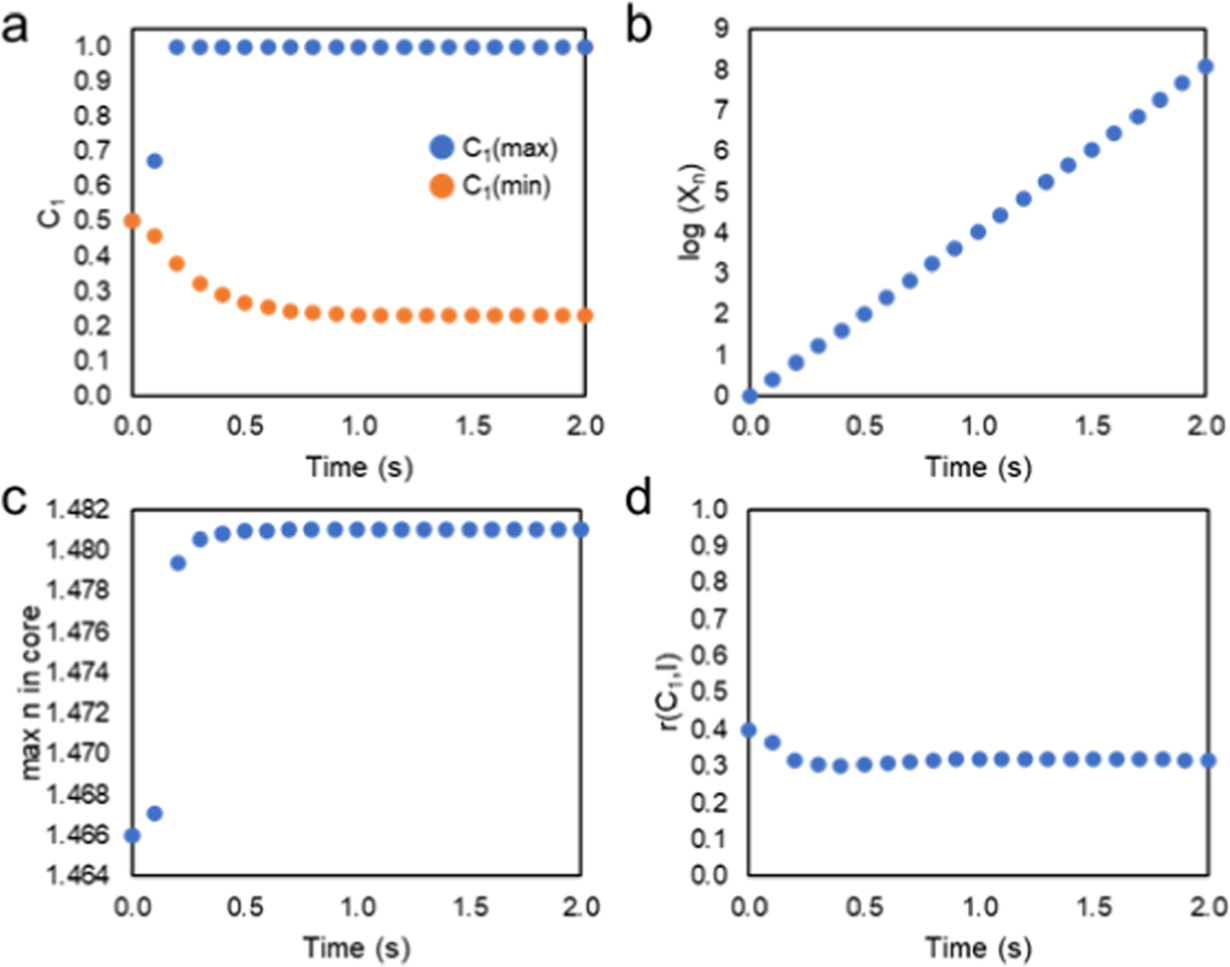
Quantitative assessment of the evolution of polymer morphology over time. (a) Maximum and minimum concentrations of *C*_1_. (b) Log of the average molecular weight. (c) Maximum refractive index in the waveguide core. (d) Spatial correlation coefficient, *r*, correlating *C*_1_ with the spatial profile of light intensity (*I*). Parameters: φ = 0.5, χ = 1, *k*_p_ = 10, *N*_2_ = 50, θ = ±30°.

Figure 2 shows the evolution of the 3D spatial distributions of the polymer concentration (*C*_1_), molecular weight (*X_n_*), optical beam intensity (*I*), and refractive index (*n*). Over the first 2 s of irradiation, the polymer morphology evolves into one in which polymer 1 (*C*_1_) becomes highly concentrated in the regions of irradiation and polymer component 2 (1 – *C*_1_) becomes rich in the nonirradiated regions. This evolution also entails an increase in the molecular weight (*X_n_*), which also spatially corresponds to the regions rich in polymer 1. The optical beams are not significantly modulated from their propagating directions, except for natural divergence, as shown and discussed for Figure 1. The refractive index in the irradiated regions also increases because of the increase in the high refractive index polymer 1 as well as the significant increase in molecular weight, in accordance with the relations to polymer volume fraction and molecular weight (eqs 1 and 3). The polymer blend and processing parameters for the evolution shown in Figure 2 result in a morphology that evolves to have a similar intersecting structure as possessed by the ensemble of intersecting optical beams. Figure 3 quantitatively tracks the evolution in the system in terms of several key parameters. First, the maximum concentration of polymer 1 (*C*_1(max)_) in the region of irradiation rapidly increases to 1 after the start of the simulation (∼0.2 s), as shown in Figure 3a. Likewise, the minimum concentration of polymer 1 (*C*_1(min)_) in the dark regions (specifically measured at the midpoint between the points of beam intersection) over time decreases, indicating an enrichment of the dark regions with polymer 2 and concurrent loss of polymer 1. The increase in the concentration of *C*_1_(max) is more immediate as it is in the region of polymerization-driven phase separation, whereas, in the dark regions, the change in concentration relies more so on polymer transport to change the concentration distribution and is thus a more gradual process. The average molecular weight of the system continually increases by orders of magnitude (Figure 3b), which indicates that the mobility of the blend also rapidly decreases. The maximum refractive index in the irradiated regions also rapidly increases to its saturable value (1.481), as shown in Figure 3c. The correlation between the phase morphology and optical beams also stabilizes at 0.3. The plots in Figure 3 indicate that the system reaches a steady state at ∼2 s, with only the molecular weight increase continuing thereafter.

**Figure 4:**
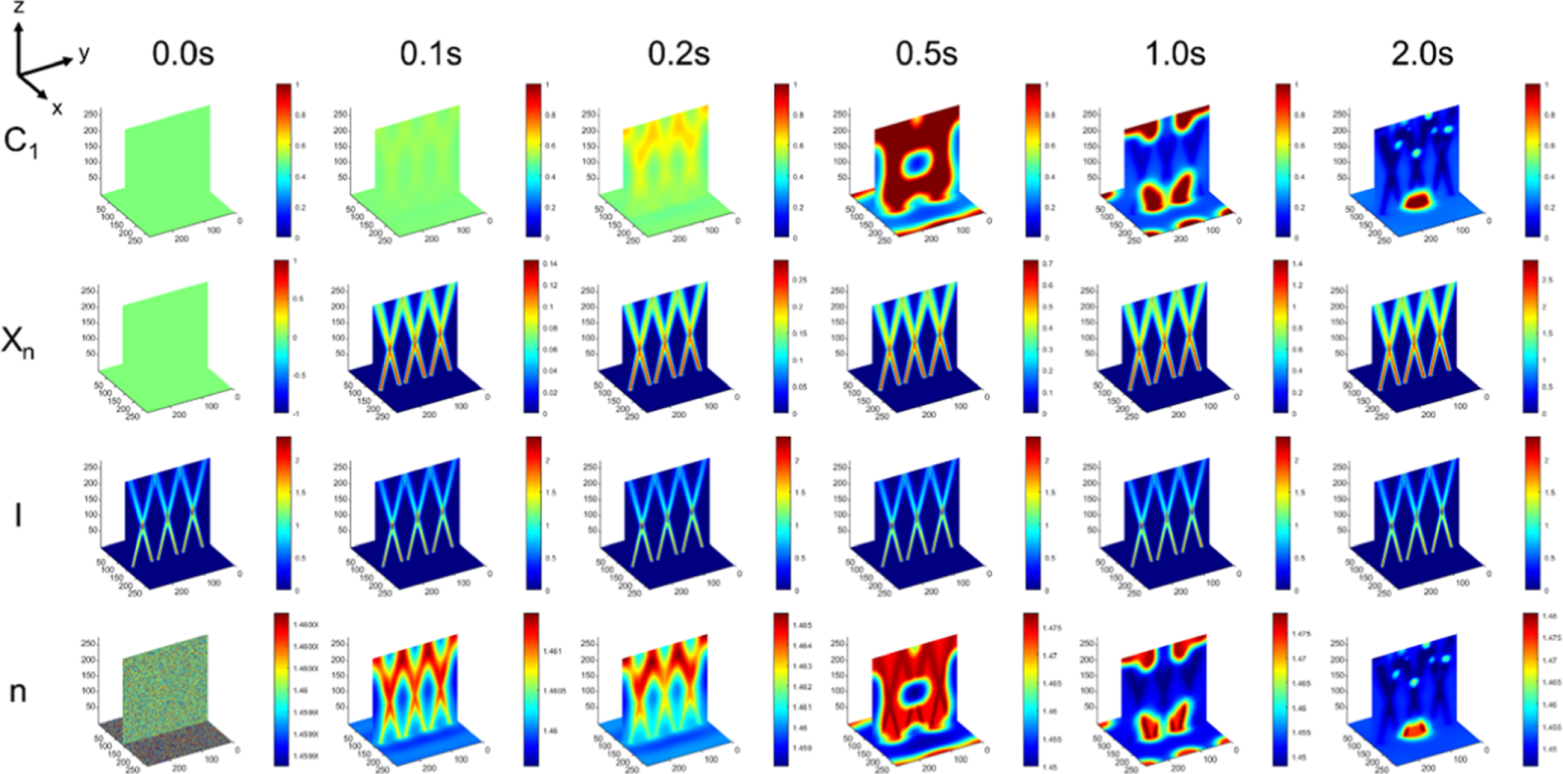
Time evolution of a system that leads to a random structure, as revealed by concentration (*C*_1_), molecular weight (*X_n_*), beam intensity (*I*), and refractive index (*n*) in the first 2 s of simulation. Parameters: φ = 0.5, χ = 1.5, *k*_p_ = 1, *N*_2_ = 50, and θ = ±15°. System selected for its lack of spatial correlation between polymer concentration and the intensity profile.

**Figure 5:**
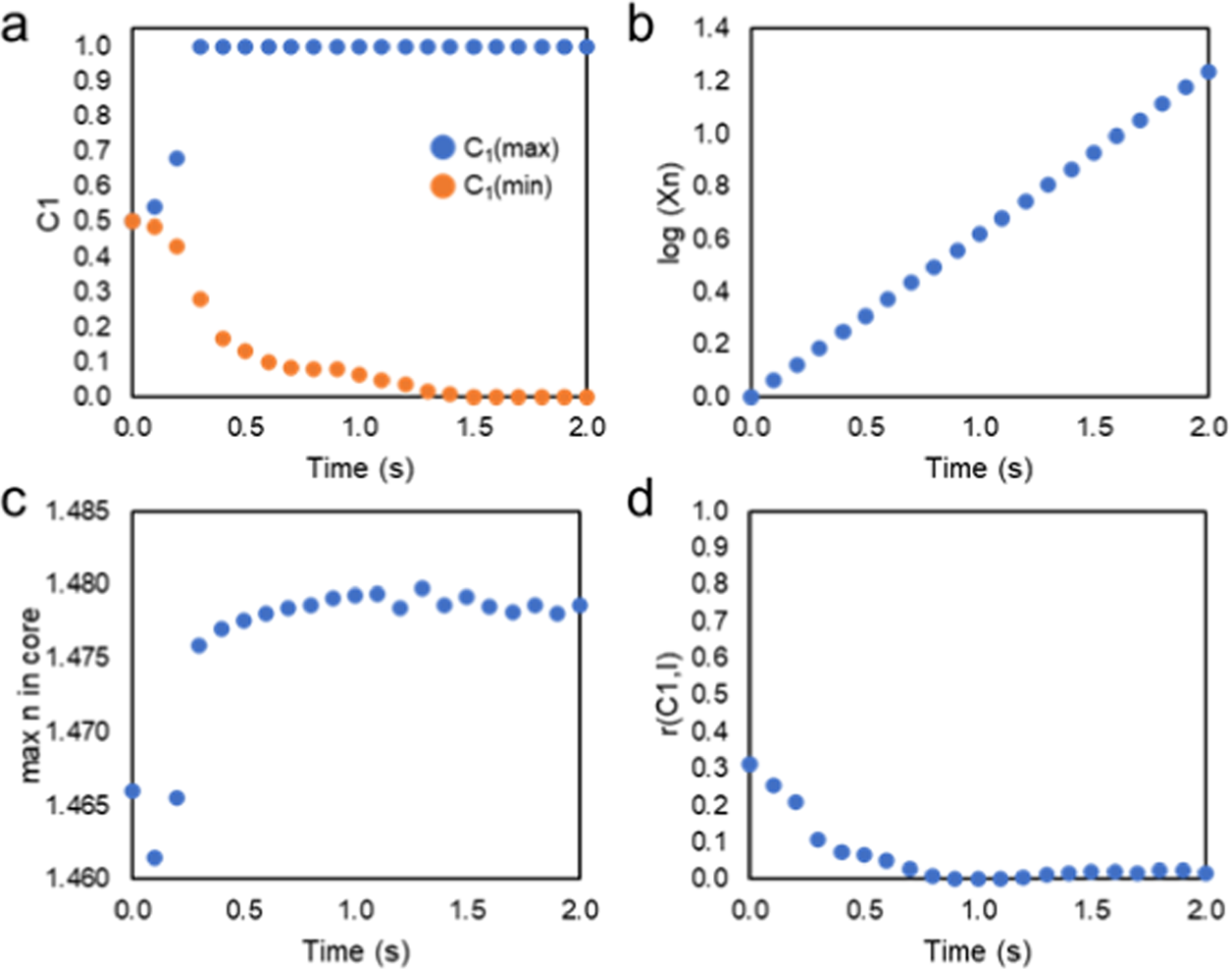
Quantitative assessment of the evolution of polymer morphology over time. (a) Maximum and minimum concentrations of *C*_1_. (b) Log of the average molecular weight. (c) Maximum refractive index in the core. (d) Spatial correlation coefficient, *r*, correlating *C*_1_ with the spatial profile of beam intensity (*I*). Parameters: φ = 0.5, χ = 1.5, *k*_p_ = 1, *N*_2_ = 50, and θ = ±30°.

Figure 4 shows the time evolution for blend and processing parameters for which the final morphology visually does not form an intersecting structure resembling that of the optical beams (in contrast to the system in Figure 2). The binary morphology as indicated by the distribution of polymer 1 is clearly random. The molecular weight is high in the region of irradiation, indicating that despite the random phase morphology, polymer 1 in the irradiated regions still reaches a large molecular weight. The optical beam intensity is not disturbed by the random phase morphology, which would indicate a robustness of the optical beam propagation to any modulations from a random binary phase morphology. Finally, the refractive index profile is also random, as expected due to the random distribution of the polymers. Both the 3D profile of the polymer distribution and the refractive index carry a small “shadow” of the intersecting structure, indicating some degree of imprinting of the pattern of the optical beam in the medium; however, a binary phase morphology and its corresponding refractive index in this case are unable to maintain this intersecting structure, owing to this set of blend parameters and processing conditions leading to random (i.e., unmitigated) phase separation. The evolution of morphology also reveals that initially there is an incipient intersecting structure; however, overtime due to the extent of the phase separation, this morphology is lost. This data would indicate that initially the photopolymerizable medium can assume the initial pattern of the intersecting beams, with subsequent phase separation determining whether it remains. In this case, the maximum concentration of polymer 1 (*C*_1(max)_) rapidly increases; likewise, the minimum concentration of polymer 1 (*C*_1(min)_) decreases to 0, indicating that this system can obtain a stronger degree of phase separation (Figure 5a) as compared to the conditions analyzed for the system presented in Figure 3a. The molecular weight continuously increases in order of magnitude (Figure 5b); however, for these conditions, particularly for a lower *k*_p_, it does not reach the same order of magnitude as for the conditions shown in Figure 3b. This would indicate that this system has a greater mobility in the first 2 s, which allows the phase separation to proceed more so and such that there is no longer any correlation between the intersecting structure of the beams and the final morphology. The system does yield a high refractive index (Figure 5c) but does not reach the maximum of 1.481, most likely owing to the lower polymerization rate constant. Consequently, the correlation between the binary phase morphology and the optical beams rapidly drops, eventually having a zero correlation (Figure 5d), which is indicative of a random morphology with no spatial resemblance to the intensity profile.

### General Dependences on Miscibility and Polymerization Rate

**Figure 6:**
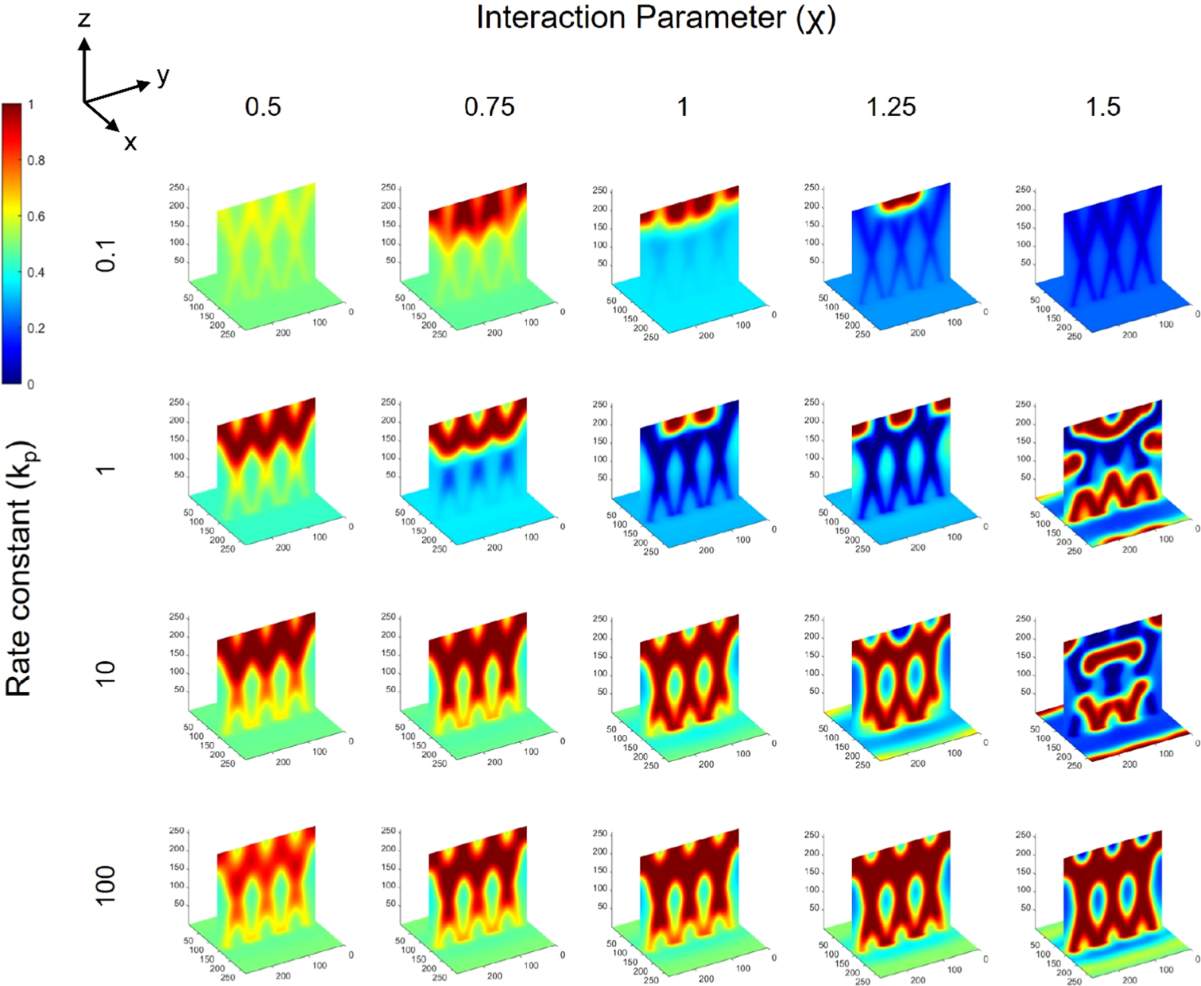
Final morphology from φ = 0.5 blends over a range of polymerization constants (*k*_p_) and interaction parameters (χ) for Δ*n* = 0.007 and *N*_2_ = 50, irradiated with ±15° parallel optical beams. Maps show rich polymer 1 regions in deeper red color and rich polymer 2 regions in deeper blue color.

Figure 6 shows image slices of the binary phase morphology produced over a mapped range of rate constants (*k*_p_) and interaction parameters (χ) for propagation angles of ±15°, whereby trends in the resultant morphology can be observed. With increasing rate constant (for any fixed χ, i.e., columns of Figure 6), an intersecting waveguide structure (cylindrical shaped phases of polymer 1) with three characteristic crossings (i.e., regions of intersection) as observed with the optical beams becomes clear. With increasing interaction parameter (for any fixed *k*_p_, i.e., rows of Figure 6), there is a greater degree of separation, which is visually apparent by the deeper red and blue regions in the 3D maps, indicating the greater presence of and deficiency in polymers 1 and 2, respectively, in the region of the optical beams (i.e., more of polymer 2 in the nonirradiated regions). What is also observed with increasing χ (for any fixed *k*_p_ value) is a loss in the apparent quality or visually apparent intersecting waveguide structure. However, the combined increase of both *k*_p_ and χ (upper left to the lower right of Figure 6) leads to a clear intersecting waveguide structure that also shows a well phase-separated morphology, consisting of intersecting cylinders rich in polymer 1 surrounded by regions rich in polymer 2. Slices that show only blue color in the 3D maps are a result of the polymer 1 random phases not being present along the position of the *yz* slice used to visualize the morphology, which cuts through the middle of the cell. Importantly, the maps show parameters whereby the binary phase morphology assumes the intersecting structure imposed by the intersecting pattern of the optical beams, namely, conditions whereby the optical beams can inscribe their pattern on morphology. Considering the converse case of slower polymerization and greater χ (lower left to the upper right of Figure 6), the apparent intersecting structure is lost, and the phase separation also evolves to form a random morphology, with reduced resemblance to the optical intensity profile. In terms of phase separation, for any polymerization rate, an increase in the χ value increases the degree of phase separation, yet only high polymerization rates preserve the intersecting structure. Likewise, for any χ value, greater polymerization rate both drives phase separation (i.e., achieving greater degrees of separation) and aids in achieving better-quality intersecting structures (i.e., resembling the optical beam pattern).

Hence, the results show that an intersecting structure and corresponding morphology are achieved through a balance in polymerization rate and blend immiscibility. Polymerization increases the molecular weight of polymer 1, which increases the thermodynamic drive for phase separation while also concurrently reducing the mobility of the polymers (emulating the increased viscosity of the system with an increase in *M_n_*). Hence, polymerization serves the purpose of both driving phase separation and slowing down the dynamics in the system, thereby constituting a tunable parameter whereby the system’s degree and extent of phase separation can be controlled. For example, high χ systems (inherently immiscible) will require high *k*_p_ values to slow down (even halt) the phase separation dynamics, so that any binary phase morphology that evolves into the intersecting structures is not lost as a consequence of continued, uncontrollable binary phase evolution. Likewise, for low χ systems (reasonably miscible), while the intersecting structure is easy to achieve, high *k*_p_ values are needed to drive phase separation (via molecular weight-based instability). The overall effects are that the higher polymerization rate ensures preservation of the intersecting structure, and combined with a high χ, the system also achieves a well phase-separated morphology in the same pattern as the structure (and of course the optical beams from which it originates). Overall, these observations show the interplay and the effect of both polymerization and miscibility and how they determine and can be used to control binary phase morphology to evolve into the same pattern as the multitude of optical beams.

### Dependence on the Optical Beam Propagation Angle

**Figure 7:**
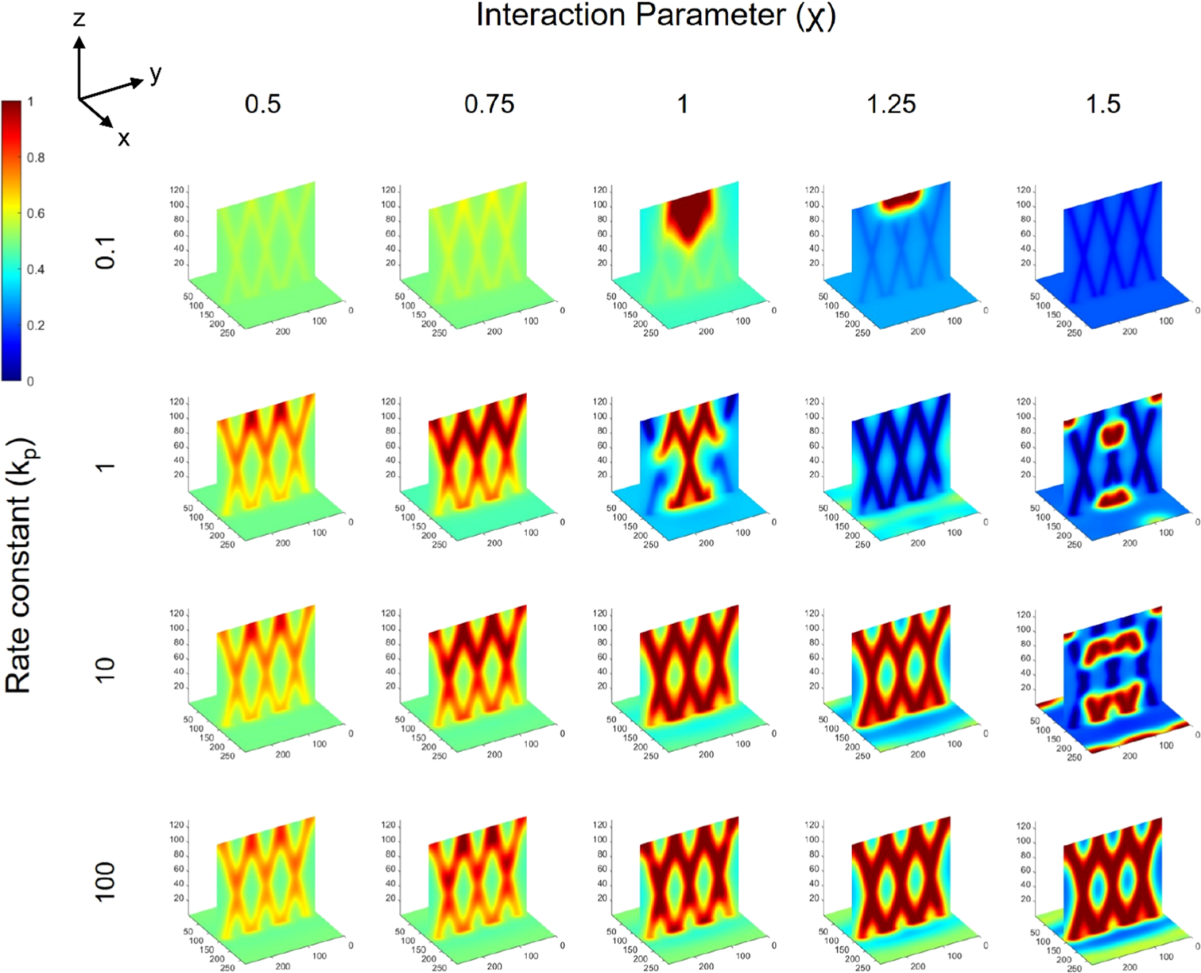
Final morphology from φ = 0.5 blends over a range of polymerization constants (*k*_p_) and interaction parameters (χ) for Δ*n* = 0.007 and *N*_2_ = 50 irradiated with ±30° parallel optical beams. Maps show rich polymer 1 regions in deeper red color and rich polymer 2 regions in deeper blue color.

The same trends with regard to the effect of polymerization rate and χ parameter can be observed for structures and morphologies formed with optical beam arrays oriented at ±30°, as shown in Figure 7. However, a slightly greater range of parameters investigated visually produced better intersecting structures vs ±15° beams. Comparison between Figures 6 and 7 shows that parameter sets, such as *k*_p_ = 1 and χ = 1 or *k*_p_ = 10 and χ = 1.25, are examples where the structures produced with ±30° optical beams show a clearer intersecting structure. This indicates that the optical beam orientation also influences the final morphology as well as the quality of the intersecting structure that may be achieved. A reason for this difference between 30 and 15° is the extent of overlap of the optical beams in the intersecting region, which is greater for the latter, which can cause the cylindrical phases composed of polymer 1 produced by each beam to begin to merge in the regions of intersection, such that their interfaces evolve as one, rather than appearing as two distinct, intersecting cylinders. This point is made clear, for example, by examining the binary phase morphologies for *k*_p_ = 100 over all χ values between ±15° beams (Figure 6) and ±30° beams (Figure 7), where the “X” shape in the intersection in the latter is visually sharper. The use of beams oriented at ±30° leads to smaller, tighter regions of intersection (i.e., smaller region of overlap), such that the resultant binary phase morphologies have clearer intersecting regions themselves.

In terms of the details of the formed lattice structures, the widths of the cylindrical polymer 1 phases are close to the width of the optical beams at lower *k*_p_. The overall volume of polymer 1 is greater than the volume of the irradiated region, such that with sufficient phase separation, the cylindrically shaped phases that intersect to form the structure can have widths that extend beyond the regions of the optical beams. Hence, the width of the phases increases with increased χ, which allows the phase separation to proceed, allowing the phases to grow. A depth dependence on the structure and morphology is also present, most noticeably at low χ values, where the degree of phase separation is greater in the deeper regions of the optical beams. Even at higher *k*_p_ values, this depth dependence remains. This variation in morphology with depth is clearly present for morphologies formed with ±15° optical beams and somewhat apparent for morphologies formed with ±30° optical beams. In addition to the obvious greater depth of propagation for the ±15° optical beams, the significant overlap and greater diffuse nature of the light intensity after their intersection can result in a greater volume of the medium being irradiated at greater depths, which can lead to phase separation proceeding to a greater extent. Whereas, closer to the entrance interface of the light, the irradiation is confined to the initial width of the optical beams, and phase separation can only proceed in those confined regions. Lack of a clear depth dependence for a propagation of ±30° optical beams is associated with the smaller region of intersection, as well as shorter depth over which the beams propagate in the simulation cell. Another notable detail in the morphologies is variations in phase separation along the cylindrical phases, with greater degrees located in the regions of intersection, which can also be explained by the greater irradiation intensity driving phase separation to a greater extent. With greater χ, these variations are reduced, and the polymer 1 phases have a relatively consistent degree of phase separation over the depth of the medium.

**Figure 8:**
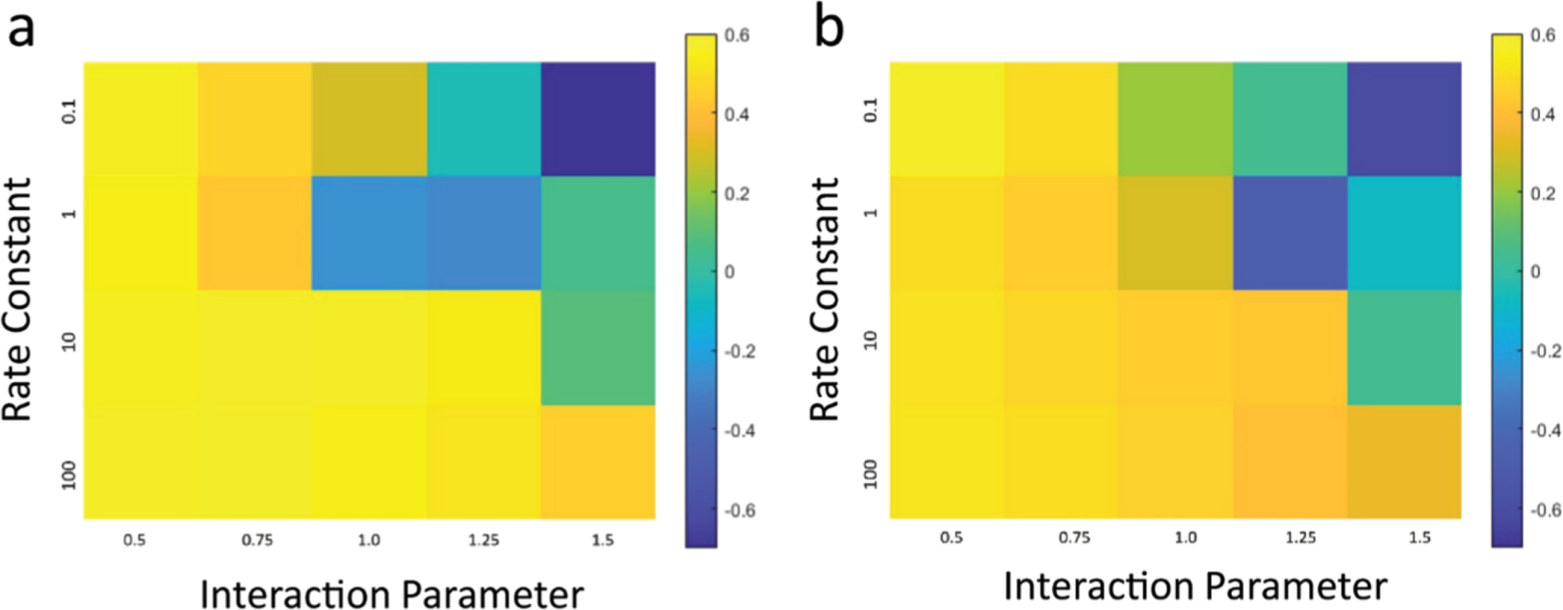
Map of the correlation coefficient values for structures produced with (a) ±15° and (b) ±30° optical beam arrays and corresponding to morphologies shown in Figures 2 and 3, respectively. Parameters: φ = 0.5, Δ*n* = 0.007, and *N*_2_ = 50.

To quantitatively assess the spatial congruency of the binary phase morphologies with optical beam patterns, a Pearson correlation coefficient was calculated for structures shown in Figures 6 (±15° optical beams) and 7 (±30° optical beams). The coefficient, *r*, was calculated specifically to examine the correlation between the 3D distribution of polymer 1 (which assumes the intersecting structure) and the optical intensity. In Figure 8, the correlation coefficient values are represented by an intensity map traced over *k*_p_ and χ, in which each colored pixel represents the *r* value corresponding to their respective structures in Figures 6 and 7. Values of *r* closer to +1 indicate polymer structures with greater spatial correlation to the optical profile. Values close to 0 indicate random morphologies (no correlation to the optical beams). Values close to −1 indicate inversion of the positions of polymers 1 and 2 or possible randomness of the binary phase morphology.

Figure 8 shows trends in the correlation values that accurately reflect and confirm visual observations of the structure and morphology over *k*_p_, χ, and beam propagation angle. For blends irradiated with either ±15 or ±30° propagating beams, positive correlations (brighter yellow) are found in the lower-left regions of the color maps, namely, higher *k*_p_ and lower χ. The positive correlations become slightly reduced with both higher *k*_p_ and χ (lower left to lower right in both Figure 8a,b). Weaker correlation and even indication of phase inversion are found in the upper right, i.e., lower *k*_p_ and higher χ. The highest correlation values are found for low *k*_p_ and low χ. Combining these *r* values with the observable richness of the phases shown in Figures 6 and 7 (indicated by the deeper red and blue regions), this confirms that with the combined increase of *k*_p_ and χ the optical beams can organize the polymer blend into well-spatially correlated structures and binary phase morphologies. Negative correlations found in the upper right corner of the parameter map (low *k*_p_ and high χ values) do correspond to either random phase separation or in some cases structures with the phases spatially inverted. However, some structures do show large negative correlation values (*r* < −0.5), which would indicate that some polymer blends resemble an intersecting structure, despite the strong presence of randomly located phases. One explanation is that phase separation proceeds quicker than structure formation because the high χ systems rapidly become thermodynamically immiscible and proceed earlier, whereafter eventually the intersecting structure emerges in the presence of this phase-separated structure. Whereas, for very high polymerization rates, the structure is rapidly instilled in the medium, allowing phase separation to then proceed without disturbing the integrity of the intersecting structure formed by polymer 1. The highest correlation for low *k*_p_ and low χ is reflective of a more incipient structure that matches well with the optical beam pattern because there is insufficient polymerization to drive phase separation. Hence, any small degree of phase separation in this case is well confined to the irradiated regions, which yields high correlation values. Overall, the correlation values confirm the presence of a balance between the two processes of polymerization and phase separation that allows both the structure and the binary phase morphology to remain congruent to the optical pattern. This balance is necessary to both allow the polymer dynamics to proceed sufficiently to gain high degrees of phase separation, before the mobility is sufficiently reduced to ensure the intersecting structure is not destroyed thereafter by excessive polymer diffusion. Comparison of the color maps between optical beam propagation angles can interestingly reveal slightly greater positive correlation coefficients for ±15°, which is evident, for example, by comparing *r* values for parameters *k*_p_ = 100 over all χ values; yet, the intersecting structure is most evident for structures formed by ±30° propagating beams. However, correlation coefficients, which are a simple mathematical approach to provide some quantification to the structure, should not be overly analyzed. Rather, their values should be generalized as reflecting structures as strongly correlating (>|0.5|) or weakly correlating (<|0.5|) as well as trends over the parameter space, without scrutinizing minute differences in the actual values. In summary, there is quantitative evidence (combined with visual observation of the structures) that specifically confirms that a wider range of blend parameters can be well organized into intersecting structures using optical beams with wider intersection angles (i.e., 30°). Hence, these values and trends in the correlation coefficients agree with the observed correlated, random, and invert morphologies in Figures 6 and 7. Note that the similar coefficient values at low *k*_p_ (i.e., 0.1 and 1) as well as their corresponding similar resultant morphologies, as shown in Figures 6 and 7, confirm that the different intensities employed for ±15 and ±30° simulations are not significant, and assessments of the effect of polymerization rate based on *k*_p_ alone are valid.

### Dependence on Other Blend Parameters

#### Molecular Weight of Polymer 2, *N*_2_

**Figure 9:**
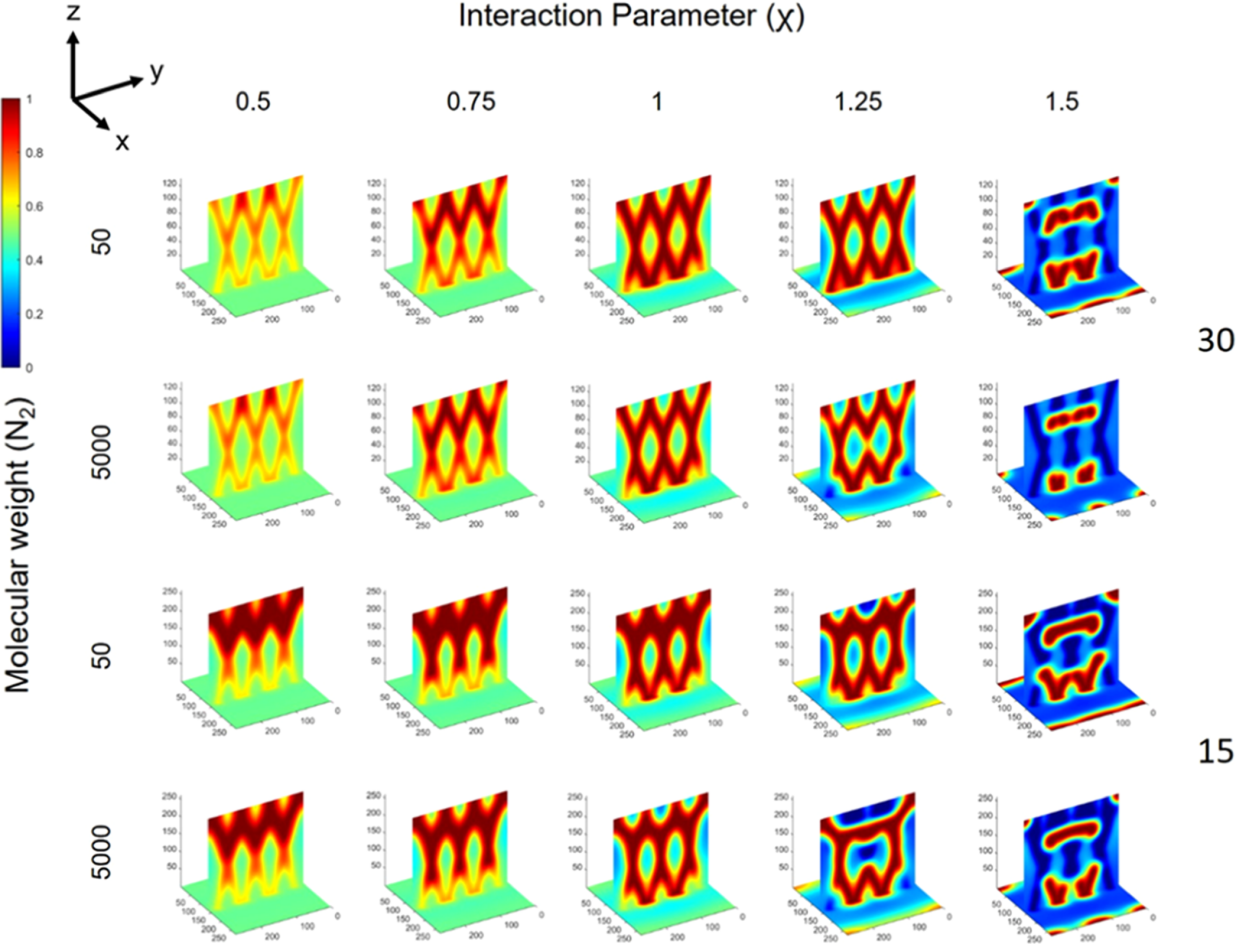
Final morphology of polymer blends obtained from φ = 0.5 and *k*_p_ = 10 and ±30° as well as ±15° optical beams over a range of χ values for *N*_2_ = 50 and 5000 and Δ*n* = 0.007. Maps show rich polymer 1 regions in deeper red color and rich polymer 2 regions in deeper blue color.

A greater molecular weight for polymer 2 has the effect of increasing the thermodynamic drive for phase separation, as revealed in Figure 9 for two different molecular weights and as a function of χ. For example, at χ 1.25, the visually observed intersecting structure when *N*_2_ = 50 appears to have the intersecting structure broken apart or warped when *N*_2_ = 5000, with a ±15° propagation angle. This is also the case for ±30° propagating beams, and intersecting structures are obtained over a greater parameter space for *N*_2_ = 50 vs *N*_2_ = 5000. This observation indicates the necessity of considering the molecular weight of the secondary component and appropriate polymerization rate (which needs to be increased for greater molecular weight polymers) to properly organize intersecting structures via mitigating the stronger thermodynamic drive for phase separation. For example, increasing the *k*_p_ from 10 to 100 resulted in there being no significant difference between the morphologies of blends with *N*_2_ = 50 and *N*_2_ = 5000 (see Figures S1 and S2, as compared to Figures 6 and 7, respectively).

#### Volume Fraction

**Figure 10:**
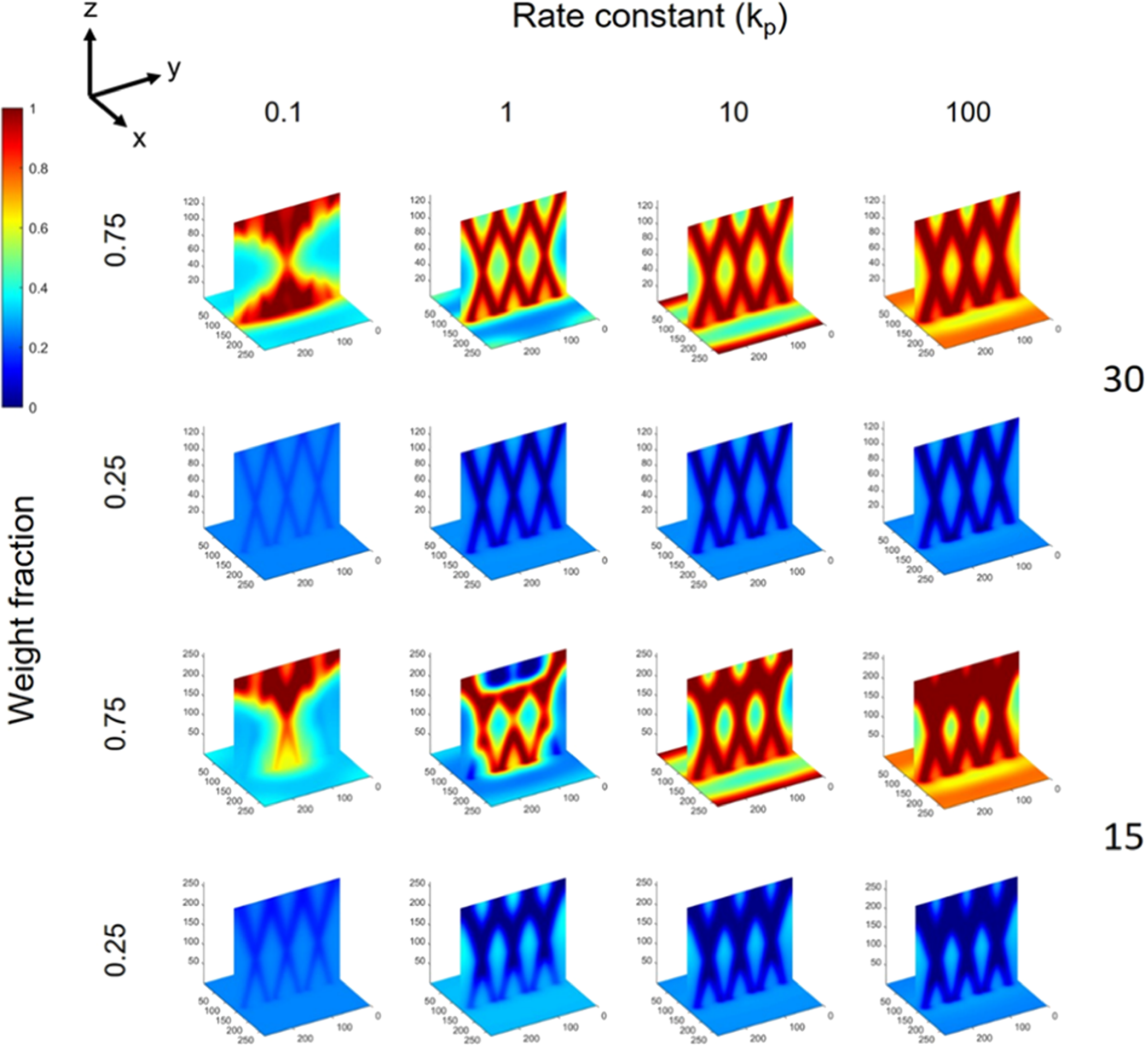
Final morphology for volume fractions φ = 0.25 and 0.75 and optical beam propagation angles ±15° and ±30°. Other parameters: Δ*n* = 0.007, *N*_2_ = 50, and χ = 1.

We examined resultant morphologies of blends with different volume fractions of polymers 1 and 2, specifically examining φ = 0.75 (more) and 0.25 (less) of polymer 1. Figure 10 shows changes in the structure and morphology for constant χ = 1 and increasing *k*_p_ for both propagation angles of ±15 and ±30°. First, intersecting structures are observed for both ±15 and ±30°, and clear intersections are observed for the latter, indicating that the angle of propagation of the optical beams has the same effect regardless of the volume fraction. Likewise, clearer intersecting structures are observed with increasing *k*_p_, once again owing to the capability of greater polymerization rate to mitigate strong phase separation. One difference between the φ = 0.25 and 0.75 blends as compared to φ = 0.5 is the greater degree of phase separation for the former. For φ = 0.75, this is indicated visually in Figure 10 by the deeper red phases in the regions of illumination, indicating richer phases as compared to the φ = 0.5 for the same χ. Likewise, for φ = 0.25, the richness of the phases is also greater as compared to φ = 0.5; however, in this case, the spatial distribution of the polymer components has inverted. This can be explained by the regions of polymerization being more thermodynamically favorable to deplete themselves of polymer 1, as a more direct pathway to phase separation (i.e., 0.25 → 0.0), rather than to become enriched (i.e., 0.25 → 1.0). This inversion can still be in accordance with our assumption that the polymerized polymer 1 remains in the irradiated region, as monomer or even low-molecular-weight molecules may still be transported out of the region to evolve the inverted morphology while leaving a low-concentration, highly polymerized intersecting structure intact in the irradiated region. In fact, the shorter concentration pathways to phase separation in both volume fractions of 0.25 and 0.75 can also explain why both result in richer binary phase morphologies. Whereas, for φ = 0.5, whether a local region is driven to expel polymer component 1 or 2 depends more on the random fluctuations in concentration gradients, which makes the phase separation dynamics initially stochastic in direction, and this effect can be exacerbated with a large enough χ value, resulting in a greater likelihood of deviations in the morphology. Comparing morphologies between φ = 0.25 and 0.75, for ±30°, both appear similar but simply inverted. However, at low *k*_p_ (i.e., 0.1), the greater content of polymer 1 (i.e., φ = 0.75) allows the binary phase morphology to extend beyond the optical pattern, both through unmitigated phase separation and the greater volume fraction of polymer 1. Whereas, for φ = 0.25, the intersecting structure remains for *k*_p_ = 0.1. This is more so the case for ±15°, where *k*_p_ values of 0.1 and 1 show loss of the intersecting structure for φ = 0.75, whereas the intersecting structure is retained for φ = 0.25. These observations point toward the reduced volume fraction of the reactive polymer as an additional tunable parameter to mitigated structure phase separation, especially at low polymerization rates. Full morphology maps for φ = 0.25 and 0.75 are provided in Figures S3–S6, all of which show similar trends over *k*_p_ and χ, as already explained herein. The correlation coefficients for φ = 0.25 and 0.75 show steady values >0.4 and only begin to drop for χ ≥ 1.25, in contrast to φ = 0.5 where there is more of a monotonic decrease with increased χ. This once again can be explained by an easier pathway toward phase separation, which allows the structure and morphology to evolve into those that are more spatially correlated to the optical pattern. Maps of the correlation coefficients can be found in Figures S7 and S8.

**Figure 11:**
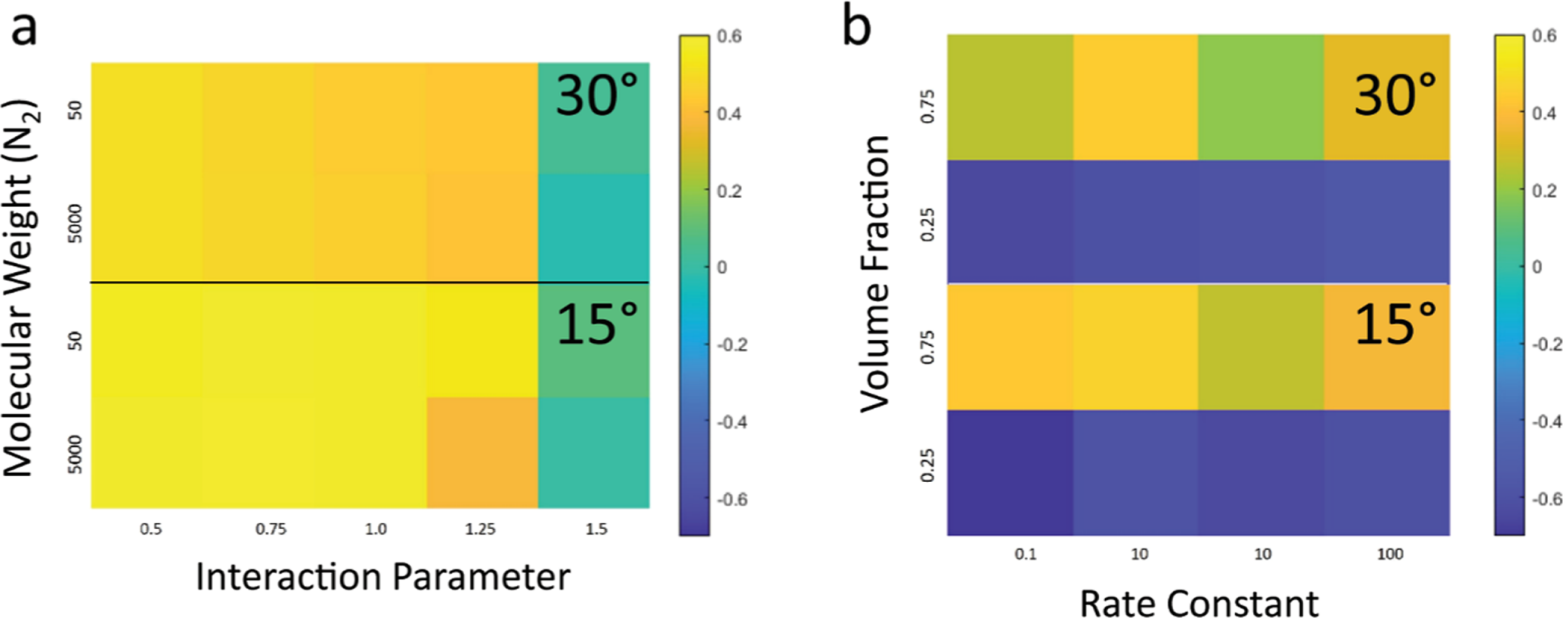
Map of the correlation coefficients for structures produced with (a) different molecular weights (*N*_2_) over χ and with *k*_p_ = 10 and (b) φ = 0.25 and 0.75 over *k*_p_ and with χ = 1 and *N*_2_ = 50. Data is divided into the respective optical beam propagation angles. Intensity maps correspond to morphologies shown in Figures 9 and 10, respectively. Other parameters: Δ*n* = 0.007.

Figure 11 shows intensity maps of the correlation coefficient as a quantitative comparison of the structural differences between *N*_2_ = 50 vs 5000 and blends φ = 0.25 vs 0.75. Figure 11a shows that smaller *N*_2_ provides slightly better spatially correlated structures over a larger value. Differences in the correlation between smaller and greater *N*_2_ are more evident with a greater χ value (owing to the combination of high *N*_2_ and χ increasing the drive for phase separation), whereas for low χ, the correlation coefficient is of similar magnitude (only slightly greater for small *N*_2_). This indicates that χ plays a stronger role in determining phase separation over the molecular weight of polymer 2 and is thus a stronger determinative parameter on the strength of the correlation between blend structure and morphology and the optical pattern. Finally, correlations are greater for a propagation angle of ±30° vs ±15° for reasons described earlier in terms of the size of the intersecting regions.

#### Refractive Index

The morphology maps show that blend and polymerization conditions can allow the system to not only form waveguide structures but also for those waveguides to reach their theoretical refractive index difference based on a pure polymer 1 as the core and polymer 2 as the cladding (Δ*n* = 1.481 – 1.446 = 0.035). Differences in the refractive index of either 1 order of magnitude greater or less than Δ*n* = 0.007 did not show any significant effect on the final structure and morphology. All optical beams underwent the same modulations, as well as the same corresponding phase-separated morphologies, along with similar correlations to *k*_p_, χ, and beam propagation angle. This is in accordance with self-trapping being possible with even very small refractive index differences (∼10^–6^), and thus index differences practically achievable for polymer systems (∼10^–3^) are well above this threshold. Hence, trends in morphology as a function of polymerization rate and χ were the same for 0.0007–0.07 (see Figures S9–S12). This finding is beneficial because reactive polymers with even the mildest refractive index change upon polymerization can be employed for the preparation of binary phase morphologies proposed herein.

Overall, with the selection of the appropriate polymerization rate, polymer blends with different χ values as well as volume fractions can be employed to form well-organized structures (with respect to the optical beams) that herein possess an intersecting lattice structure. The polymerization rate not only drives phase separation but can also mitigate strong phase separation dynamics (via molecular weight-dependent mobility) so that phase separation does not cause the morphology to deviate from resembling the pattern of the optical intensity profile. With smaller intersecting angles, there is the possibility for the evolving cylindrical phases to become merged and cause further deviation in morphology at greater depths; however, this effect can once again be mitigated by increasing the polymerization rate. Reducing the volume fraction of the reactive component can also be used to mitigate strong phase separation, especially at low polymerization rates, to achieve better structures. Experimentally, the polymerization rate would be adjusted via photoinitiator as well as light intensity.^57,60^ Adjusting the volume fraction is also a parameter to enable well-ordered structures to appear, and inversion of the phases is also possible. While higher polymerization rates enable control of the phase separation, it can come at the expense of the richness in the phases, especially for low χ systems. All well-organized structures are essentially multiwaveguide lattices by virtue of their core-cladding architecture, which shows that this is a method to produce new types of material structures with possibly interesting optical transmission properties, which will be explored in future work. The intersecting, net-like structure may also open opportunities in other applications, including tunable mechanical properties, transport, and even microfluidics, filtration, and detection (with an etchable polymer phase), which may be pursued via photopolymerization of blends using transmitted optical beams.

## Conclusions

We have simulated the formation of structure and binary phase morphology in photoreactive polymer blends irradiated with two sets of intersecting optical beam arrays. The results indicate that in addition to the polymer blend parameters (e.g., χ) and polymerization rate (*k*_p_), the orientations of the beams play a role in determining the quality of the organized structures. With the aim of obtaining polymer blend morphologies consistent with the pattern of optical beams, the polymerization rate can be tuned to help mitigate the phase separation dynamics. Likewise, for smaller beam orientation angles, in which regions of larger overlap can drive phase separation to deviate from the pattern of the beams, polymerization rate can also mitigate this effect to obtain well-defined structures. Polymerization rate as a tunable parameter shows the capability to help control the polymer blend morphology over a range of χ values. This work theoretically demonstrates the capability to pattern and organize polymer blends using arrays of optical beams through control of the polymerization kinetics and blend parameters toward new polymer materials that offer new structure–property relationships.

## Supporting Information Available

Final morphologies mapped over *k*_p_ and χ for other values of *N*_2_ and Δ*n* (PDF)
