# Supplementary material for: Simulations of Structure and Morphology in Photoreactive Polymer Blends under Multibeam Irradiation
Source: J Phys Chem C Nanomater Interfaces. 2022 Apr 6;126(15):6700–15. doi: 10.1021/acs.jpcc.1c09993 (PMC9037196; doi:10.1021/acs.jpcc.1c09993)
Supplement: Supplementary file 1 — jp1c09993_si_001.pdf [file jp1c09993_si_001.pdf]

Simulations of Structure and Morphology in Photoreactive Polymer Blends Under Multi-Beam Irradiation

Nannan Ding, Ian D. Hosein*

1. Syracuse University, Department of Biomedical and Chemical Engineering, Syracuse, NY, 13244

* Corresponding Author: idhosein@syr.edu

**Polymer Blend Morphology for Higher Molecular Weights**

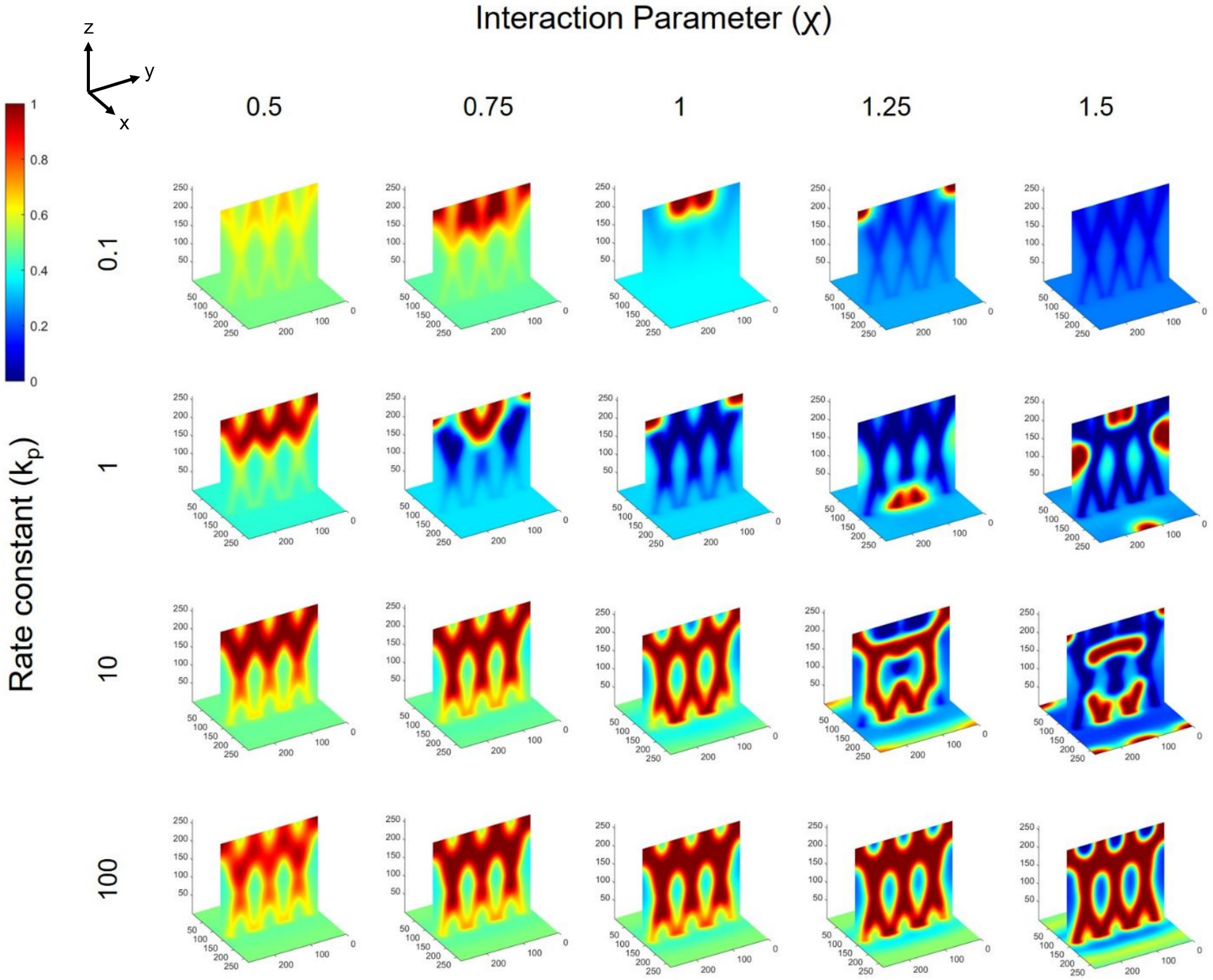


Figure S1. Final morphology from φ = 0.5 blends over a range of polymerization constants ($k_p$) and interaction parameters ($\chi$) for refractive ind

ex of 0.007 and $N_2$ of 5000 with ±15° parallel optical beams.

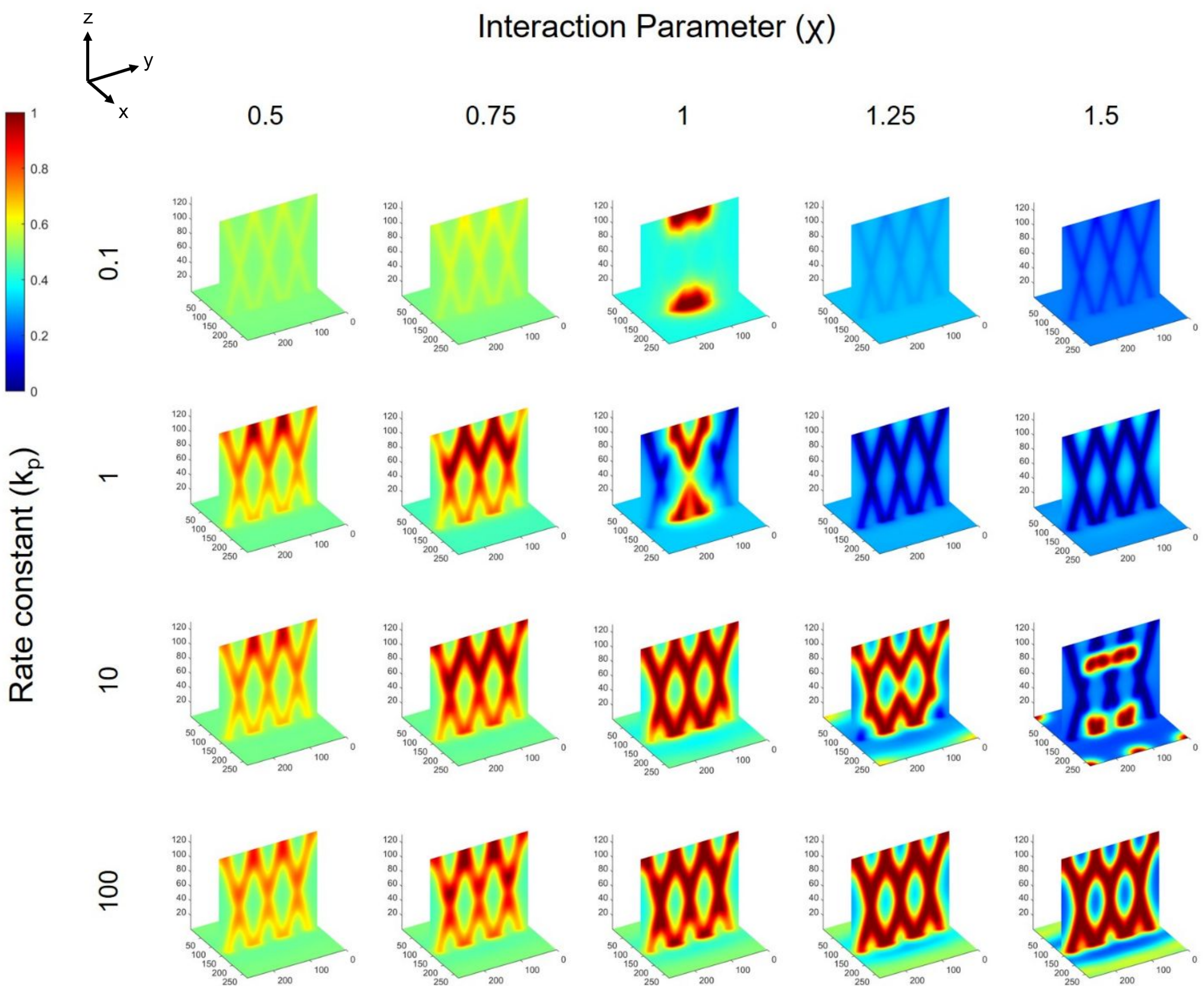


Figure S2. Final morphology from φ = 0.5 blends over a range of polymerization constants ($k_p$) and interaction parameters ($\chi$) for refractive index of 0.007 and $N_2$ of 5000 with ±30° parallel optical beams.

**Variation of Volume Fraction**

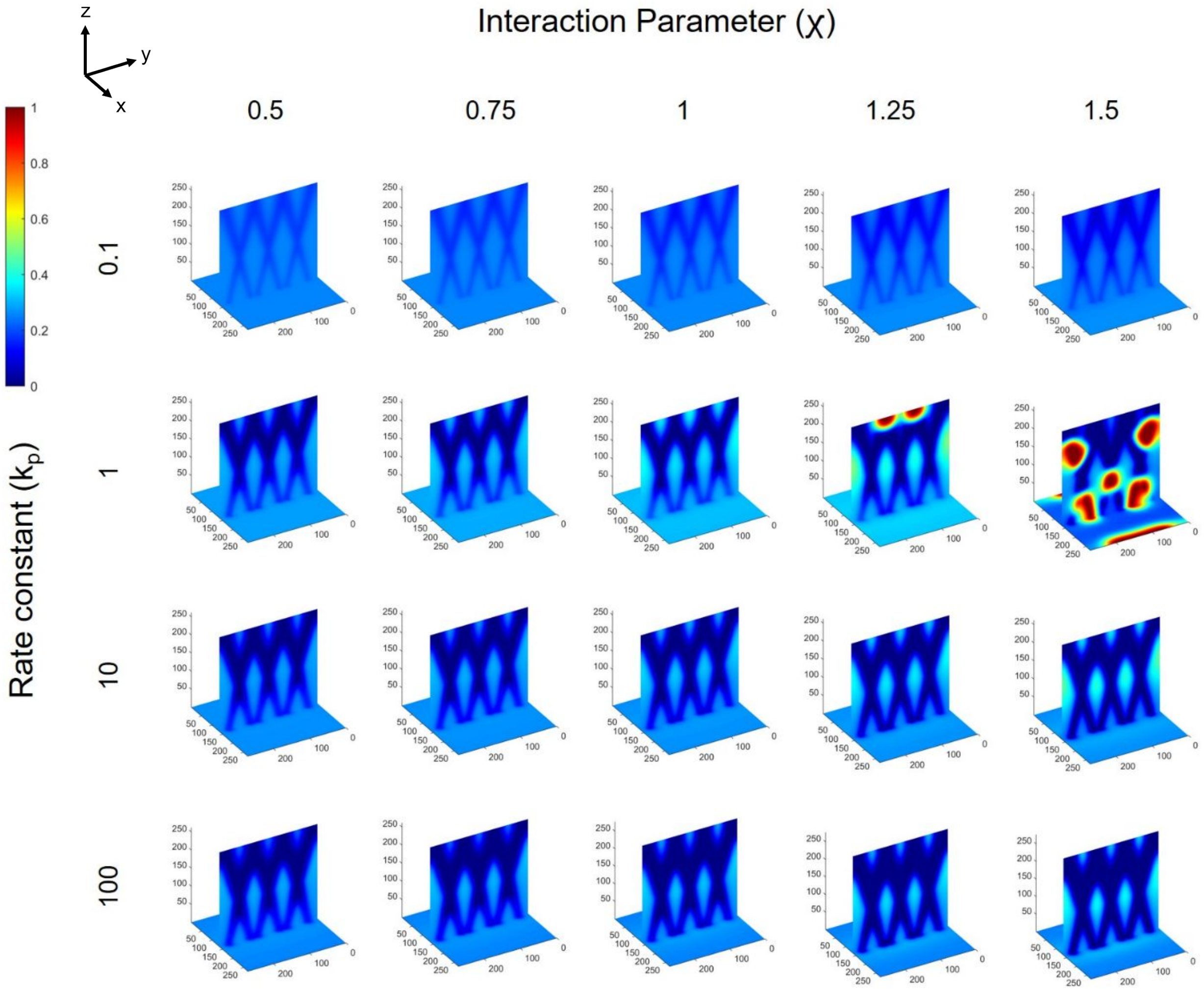


Figure S3. Final morphology from φ = 0.25 blends over a range of polymerization constants ($k_p$) and interaction parameters ($\chi$) for refractive index of 0.007 and N2 of 5000 with ±15° parallel optical beams.

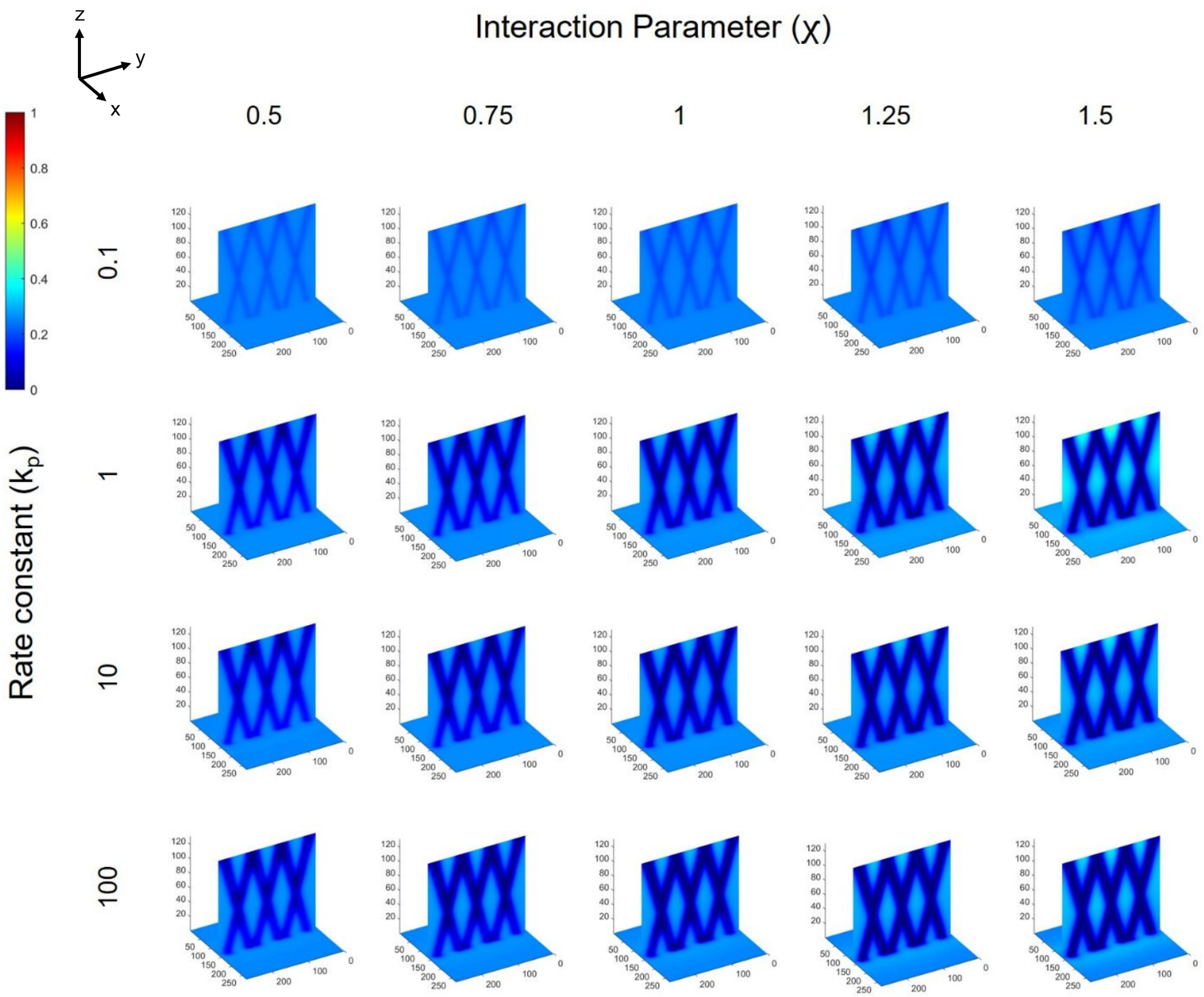


Figure S4. Final morphology from φ = 0.25 blends over a range of polymerization constants ($k_p$) and interaction parameters ($\chi$) for refractive index of 0.007 and N2 of 5000 with ±30° parallel optical beams.

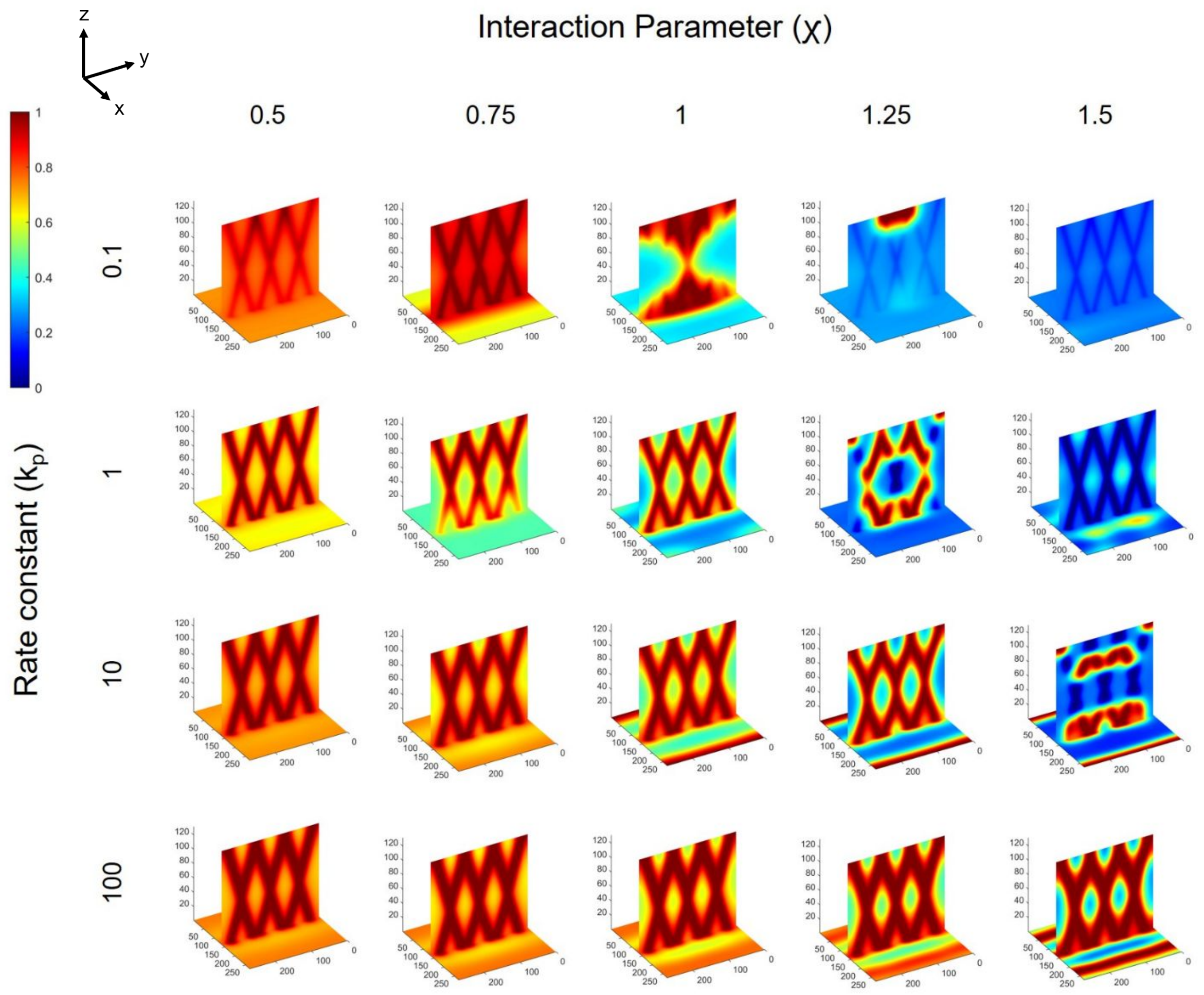


Figure S5. Final morphology from φ = 0.75 blends over a range of polymerization constants ($k_p$) and interaction parameters ($\chi$) for refractive index of 0.007 and N2 of 5000 with ±15° parallel optical beams.

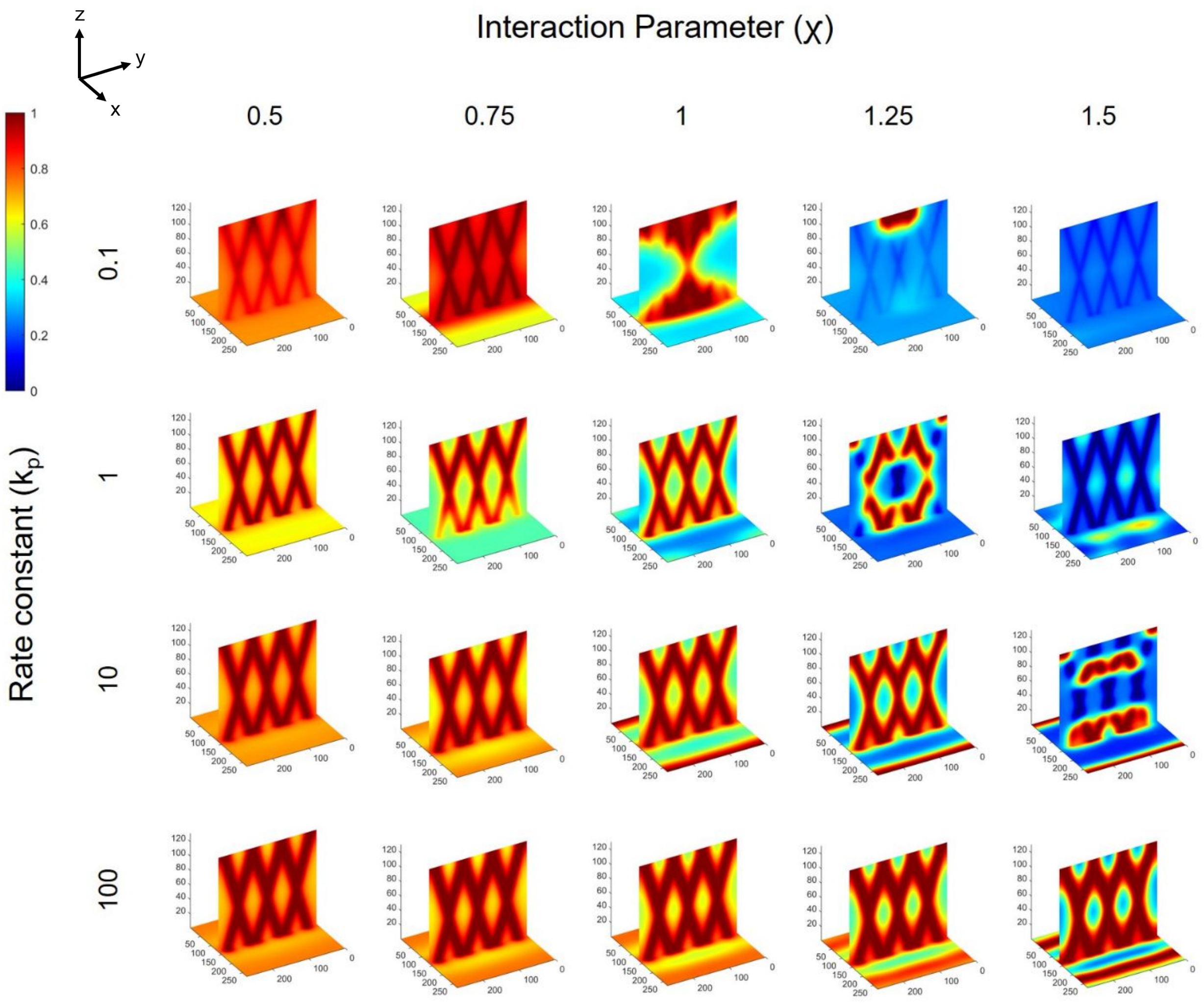


Figure S6. Final morphology from φ = 0.75 blends over a range of polymerization constants ($k_p$) and interaction parameters ($\chi$) for refractive index of 0.007 and N2 of 5000 with ±30° parallel optical beams.

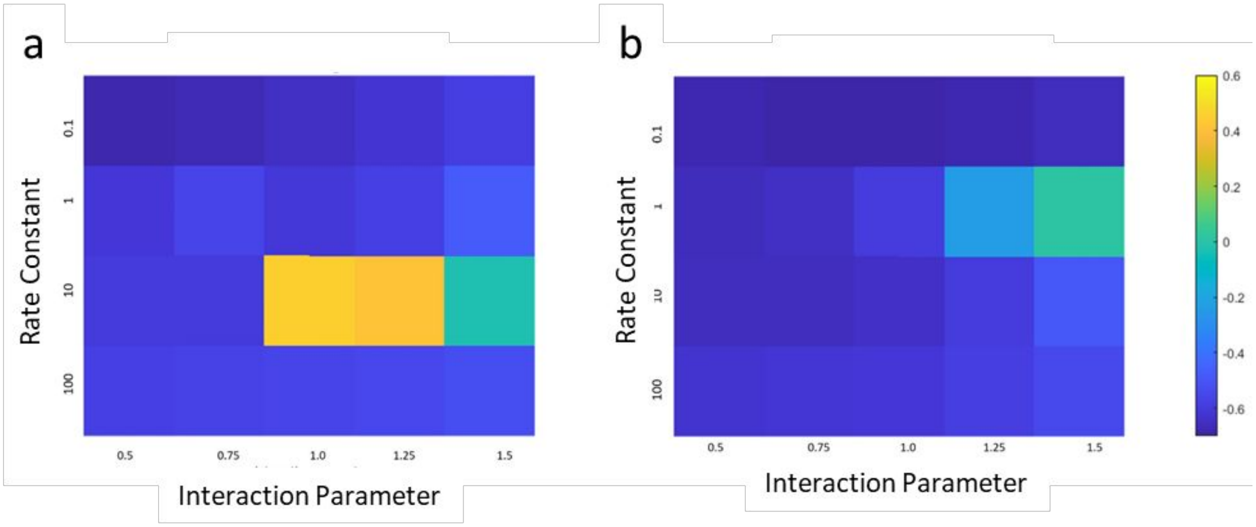


Figure S7. Map of the correlation coefficient values for structures produced from φ = 0.25 blends with (a) ±30° and (b) ±15° optical beam arrays. Other parameters: $\Delta n = 0.007$ and $N_2 = 50$.

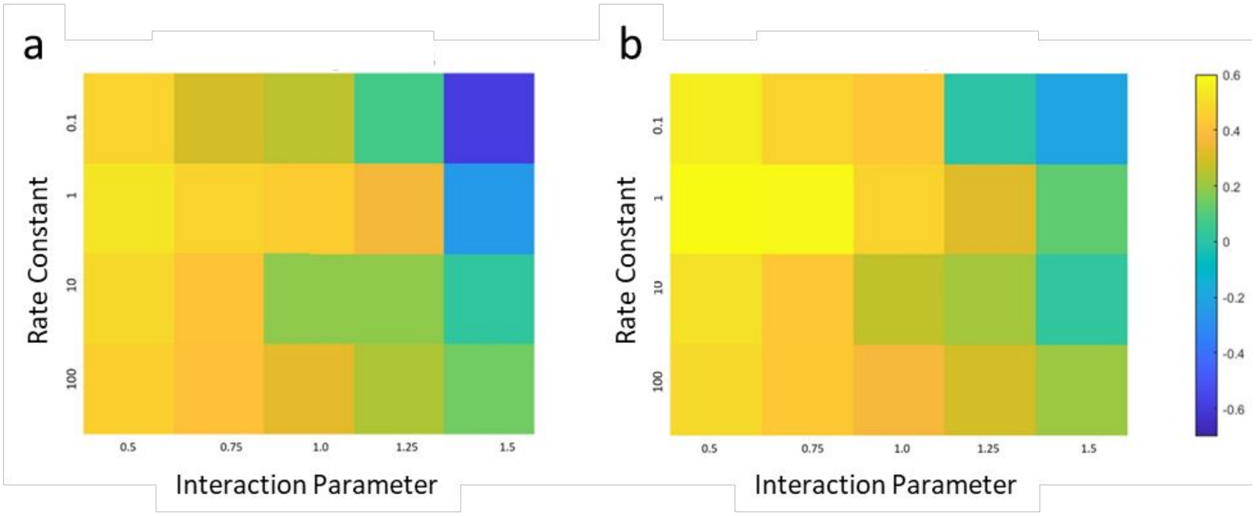


Figure S8. Map of the correlation coefficient values for structures produced from φ = 0.75 blends with (a) ±30° and (b) ±15° optical beam arrays. Other parameters: $\Delta n = 0.007$ and $N_2 = 50$.

**Variation of Refractive Index Difference**

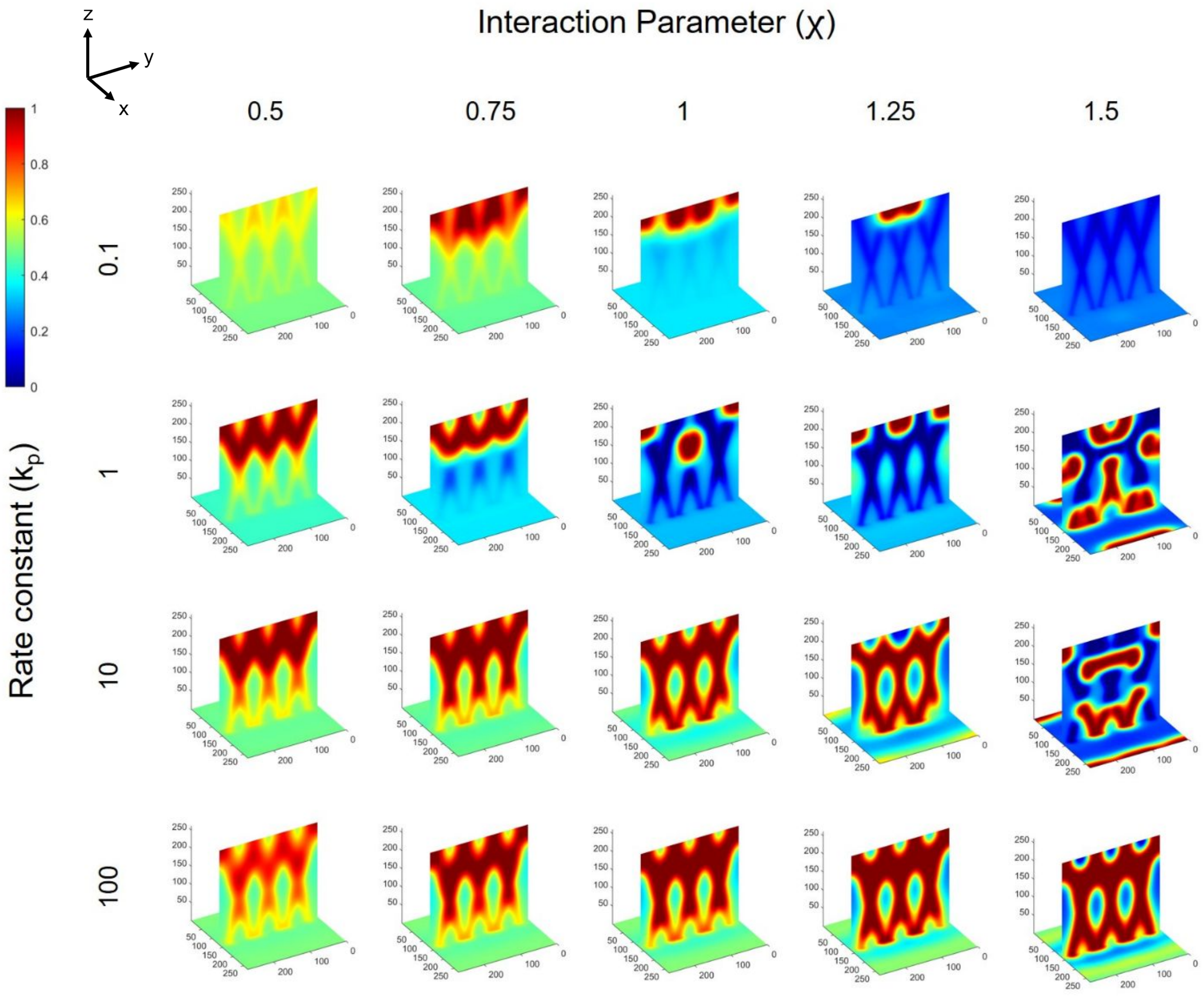


Figure S9. Final morphology from φ = 0.5 blends over a range of polymerization constants ($k_p$) and interaction parameters ($\chi$) for refractive index of 0.0007 and $N_2$ of 50 with ±15° parallel optical beams.

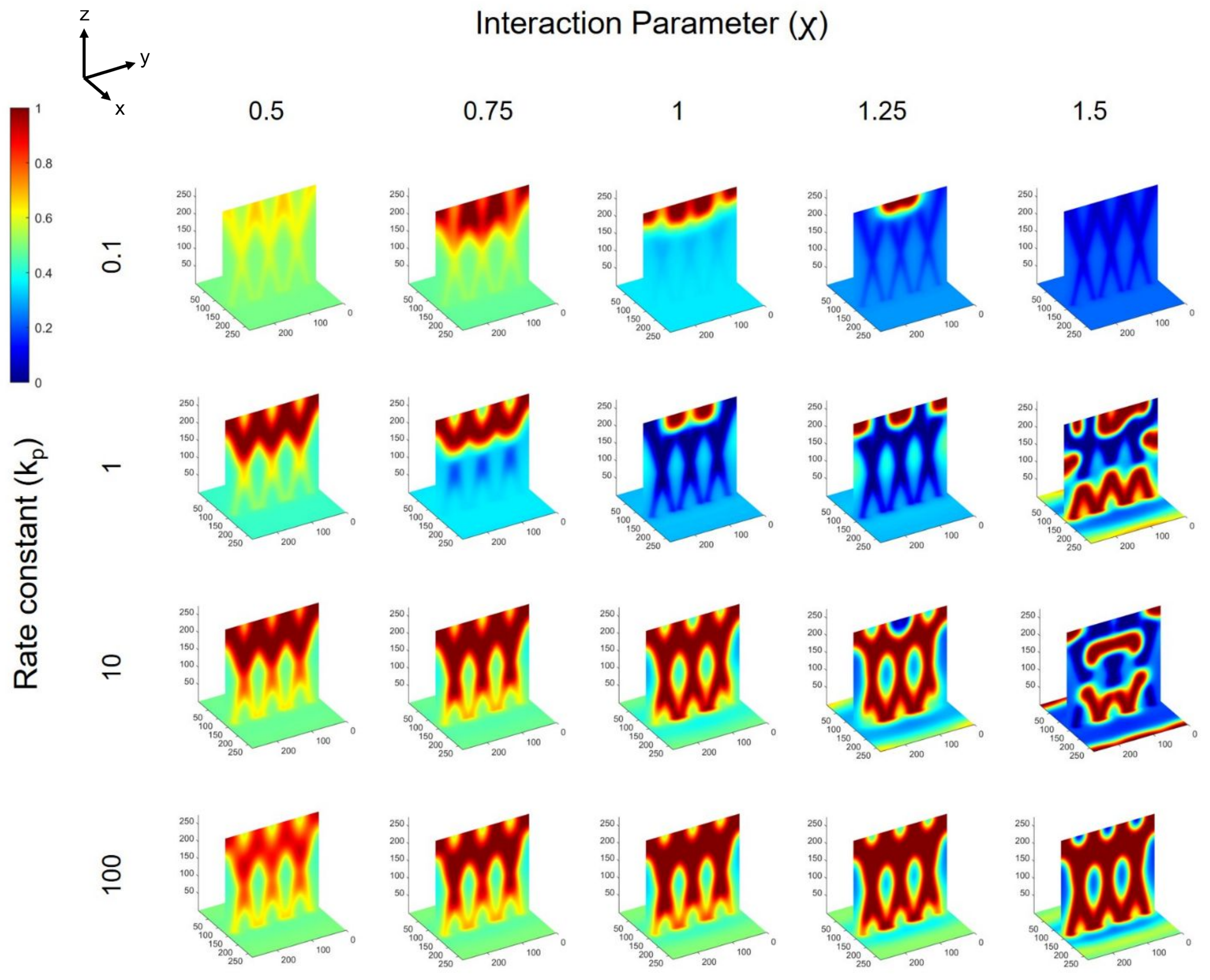


Figure S10. Final morphology from φ = 0.5 blends over a range of polymerization constants ($k_p$) and interaction parameters ($\chi$) for refractive index of 0.07 and $N_2$ of 50 with ±15° parallel optical beams.

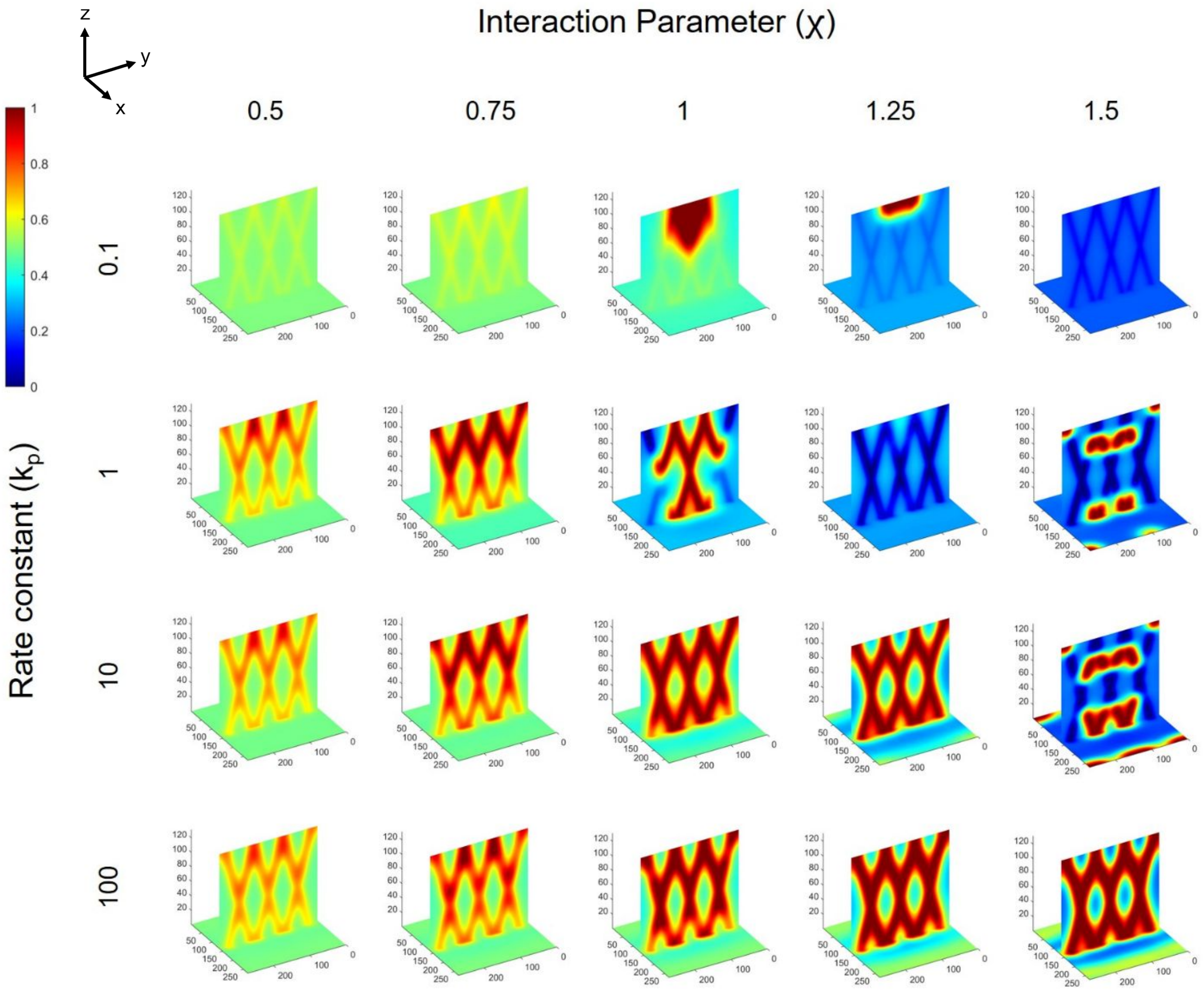


Figure S11. Final morphology from φ = 0.5 blends over a range of polymerization constants ($k_p$) and interaction parameters ($\chi$) for refractive index of 0.0007 and $N_2$ of 50 with ±30° parallel optical beams.

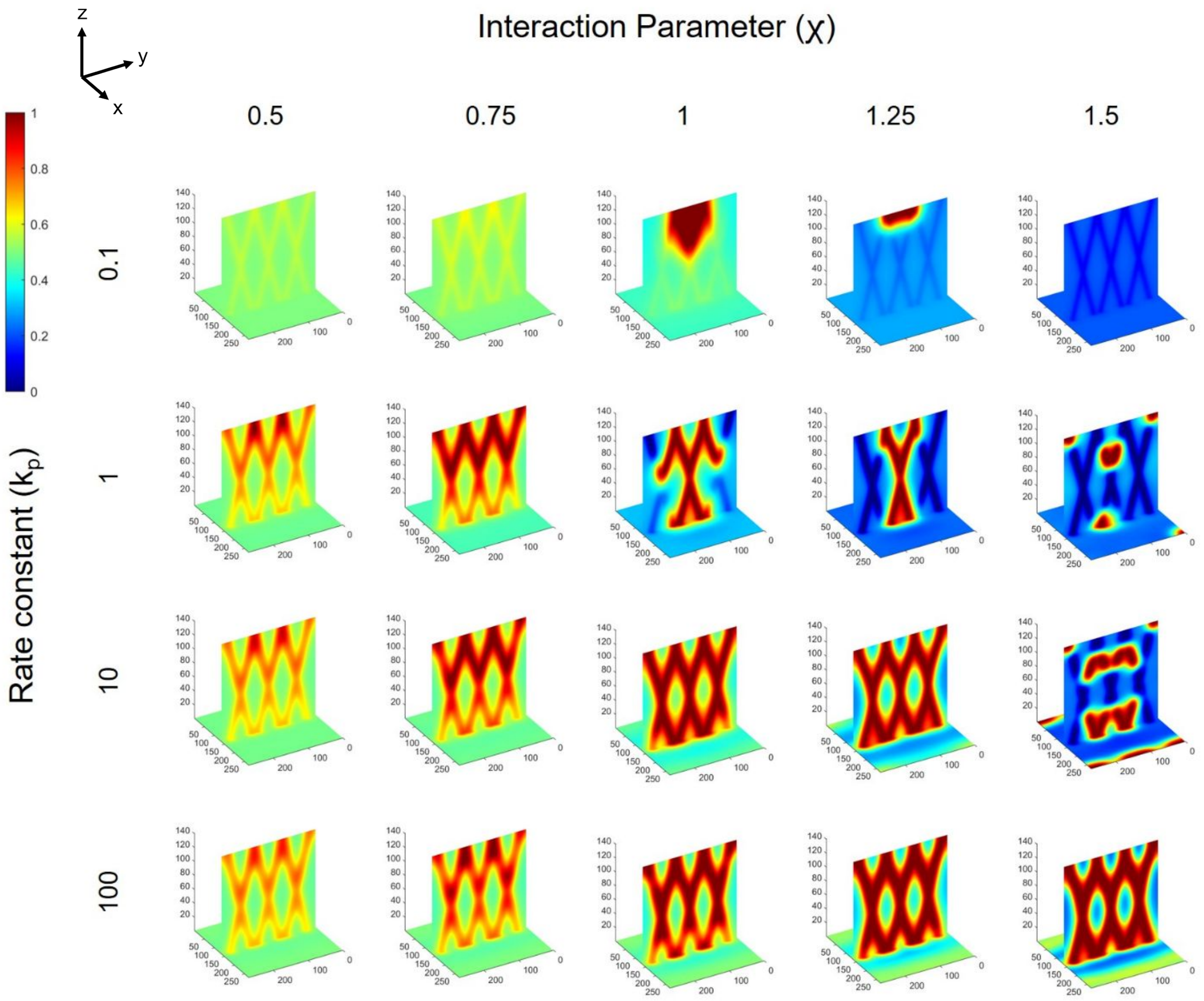


Figure S12. Final morphology from φ = 0.5 blends over a range of polymerization constants ($k_p$) and interaction parameters ($\chi$) for refractive index of 0.07 and $N_2$ of 50 with ±30° parallel optical beams.
